# Adjusted Indirect and Mixed Comparisons of Drug Therapy for Neuropathic Cancer Pain

**DOI:** 10.1155/prm/9970065

**Published:** 2026-08-30

**Authors:** Ping Huang, Tingting Nan, Ruping Ni, Yuhong Huang, Maobai Liu, Jing Yang

**Affiliations:** ^1^ Department of Pharmacy, Fujian Medical University Union Hospital, Fuzhou City, Fujian Province, China, fjmu.edu.cn; ^2^ Department of Pharmacy Management, Nanping First Hospital Affiliated to Fujian Medical University, Nanping City, Fujian Province, China, fjmu.edu.cn; ^3^ The School of Pharmacy, Fujian Medical University, Fuzhou, Fujian, China, fjmu.edu.cn; ^4^ Xiamen Haicang Hospital, Xiamen, Fujian, China; ^5^ Department of Clinical Pharmacy, Nanping First Hospital Affiliated to Fujian Medical University, Nanping City, Fujian Province, China, fjmu.edu.cn

**Keywords:** antidepressants, antiepileptics, cancer pain, network meta-analysis, neuropathic cancer pain, NSAIDs, opioids, plant-derived drugs, systematic review

## Abstract

**Objectives:**

Treatments for neuropathic cancer pain (NCP) are often based on guidelines or treatment data for neuropathic pain of noncancer origin. Therefore, their relative efficacy and safety are not well characterized and should be determined. This study aimed to compare the efficacy and safety of various pharmacological interventions for NCP.

**Methods:**

We systematically searched PubMed/MEDLINE, Embase, and the Cochrane Central Register of Controlled Trials for relevant randomized controlled trials (RCTs) from inception to June 13, 2024; 36 studies were included. The primary outcome was pain intensity; secondary outcomes included the effective pain relief rate, nausea and vomiting, and overall adverse events. The certainty of evidence was assessed using the CINeMA web application.

**Results:**

The combination of opioids, antiepileptics, and nonsteroidal anti‐inflammatory drugs (NSAIDs) appeared to be the most effective treatment to reduce pain intensity (surface under the cumulative ranking [SUCRA] curve: 0.91), followed by the combination of antiepileptics and antidepressants (SUCRA: 0.90). For secondary outcomes, the combination of opioids and neurotropin was associated with the highest probability of achieving the best pain relief rate (SUCRA: 0.81), whereas vitamin B12 appeared to be relatively ineffective. Regarding safety, vitamin B12 was associated with the highest probability of being the safest treatment for nausea and vomiting (SUCRA: 0.90), followed by antiepileptic drugs (SUCRA: 0.87). For overall adverse events, antidepressants were associated with the highest probability of being the safest treatment (SUCRA: 0.86). For the primary outcome, 84.8% of the comparisons were rated as being of low or very low confidence according to the CINeMA method.

**Conclusions:**

Antiepileptics and antidepressants, alone or in combination, and certain opioid‐based combinations may be effective for NCP based on current evidence, although the certainty of this evidence is low. Some plant‐derived compounds also demonstrated potential analgesic effects, although these findings are exploratory and based on a limited number of trials, and further evidence is needed to verify their efficacy. Given the low certainty of most comparisons, clinicians should interpret these results cautiously and balance potential benefits with safety profiles when selecting treatments for NCP.

## 1. Introduction

With advances in cancer treatment, the number of long‐term survivors continues to increase. However, cancer‐associated pain remains a prevalent issue. More than a third of patients with cancer pain experience neuropathic cancer pain (NCP) [1], which is caused by nerve injury, tumor‐related compression, or anticancer therapies such as chemotherapy and radiotherapy. NCP is characterized by burning, tingling, numbness, stabbing, or pricking sensations [2]. This condition responds poorly to analgesics, is challenging to manage, and significantly impairs daily function and quality of life [3].

Tricyclic antidepressants, serotonin–noradrenaline reuptake inhibitors (particularly duloxetine), pregabalin, and gabapentin are first‐line treatments for neuropathic pain [4–6]. The National Comprehensive Cancer Network Guidelines for Adult Cancer Pain (Version 1.2020) also recommend antidepressants and anticonvulsants as first‐line adjuvant analgesics for NCP. However, the adoption of these treatments in cancer patients is often based on guidelines or therapy data pertaining to neuropathic pain of noncancer origin [7], likely due to the paucity of clinical data specific to NCP. Previous meta‐analyses and systematic reviews on NCP have yielded conflicting results [8–10] or failed to provide clear conclusions due to the limited number of included studies [11, 12]. Moreover, traditional meta‐analyses evaluate only direct, pairwise comparisons between therapeutic agents. Previous studies have largely focused on antidepressants and antiepileptics, and the number of randomized controlled trials (RCTs) on specific drugs for NCP remains limited. Network meta‐analysis (NMA) integrates all available direct and indirect evidence across multiple trials, allowing for the comparison of various therapeutic regimens [13]. The present NMA aimed to comprehensively evaluate the efficacy and safety of multiple therapeutic regimens for NCP using data from RCTs.

## 2. Methods

This meta‐analysis adhered to the Preferred Reporting Items for Systematic Reviews and Meta‐Analyses extension statement for reporting systematic reviews incorporating NMA [14] (Supporting Table S1) and was registered with the International Prospective Register of Systematic Reviews (CRD42021254302).

### 2.1. Data Sources and Searches

The PubMed/MEDLINE, Embase, and Cochrane Central Register of Controlled Trials databases were searched for relevant RCTs from inception to June 13, 2024, using a targeted search strategy (Supporting Table S2). No language restrictions were applied. Reference lists of eligible trials, relevant reviews, and guidelines were also searched.

### 2.2. Study Selection

Two co‐authors independently screened the titles, abstracts, and full‐text articles of potentially eligible studies, resolving disagreements through discussion. The inclusion criteria were as follows: (a) adult patients (age ≥ 18 years) diagnosed with NCP (related to cancer or cancer treatment); (b) systemic or local intervention (oral, transdermal, intravenous, or subcutaneous routes); and (c) RCTs. The exclusion criteria were as follows: (a) studies on neuropathic pain not related to cancer (such as painful diabetic neuropathy, postherpetic neuralgia, and trigeminal neuralgia); and (b) case studies and series, animal studies, conference abstracts and summaries, reviews and meta‐analyses, or any other article category other than RCTs.

### 2.3. Data Extraction and Quality Assessment

Data extraction and risk‐of‐bias assessment were conducted by a single author using MS Excel, with verification of the extracted data by a second author. Two authors independently obtained relevant data from eligible trials. Disagreements were resolved by rechecking the original article, followed by discussion. The risk of bias for each included study was assessed using the Cochrane risk‐of‐bias tool (RoB 2.0) [15]. The extracted information included study characteristics (first author, country, title, publication date, and study design); patient characteristics (age and sex); intervention details (treatment types, adjuvant drugs, follow‐up duration, number of participants per arm, and pain scale); outcomes (pain intensity, proportion of patients with pain relief, and adverse events); and other relevant information.

For trials with different follow‐up periods, data for the longest follow‐up within 3 months were extracted, as only two studies had follow‐up periods exceeding 3 months. For crossover RCTs, only data from the first period were extracted to avoid potential carryover effects. Missing data, such as standard deviations, were estimated from published data [16]. When the mean was unavailable, the median was used instead. Intent‐to‐treat data were used when available.

### 2.4. Quality of Evidence Assessment

The certainty of evidence for each outcome was evaluated using the framework described by Salanti et al. [17] and implemented using the CINeMA web application. Certainty of evidence was graded as high, moderate, low, or very low [18]. For all outcomes, we examined the certainty of evidence for each comparison across six domains: within‐study bias, across‐study bias, indirectness, imprecision, heterogeneity, and incoherence. Intra‐study bias and indirectness were evaluated using a percentage contribution matrix. Imprecision, heterogeneity, and inconsistency were evaluated by considering their effects on clinical decision‐making.

### 2.5. Outcomes

The primary outcome was the change in pain intensity from baseline to endpoint, assessed using scales that could be linearly transformed to a 10‐point scale. All pain intensity measures were converted to 10 cm Visual Analog Scales (VASs) using linear transformation, assuming shared measurement properties across instruments [19]. The effect size (mean difference [MD]) was calculated as the difference in pain intensity between active treatment (or medication class) and placebo (or a different active treatment or medication class). A negative MD indicated improved pain relief.

Secondary outcomes included the effective pain relief rates, nausea and vomiting, and the total number of adverse events in each arm. The effective pain relief rate was defined as the proportion of patients achieving at least 50% less pain than the levels reported at baseline.

### 2.6. Assessment of Clinical Assumptions

Transitivity, a fundamental assumption of NMA, requires that patient and study characteristics across trial groups be sufficiently similar for indirect comparisons [20]. Confidence in transitivity was assessed by qualitatively evaluating the distribution of the potential effects of correction factors for different direct comparisons [21].

### 2.7. Statistical Analysis

A frequentist framework was used for the NMA. Dichotomous outcomes are reported as relative risks (RRs) with 95% confidence intervals (CIs), and continuous outcomes as MDs with 95% CIs. Statistical heterogeneity between studies was analyzed using Cochran’s *Q* test and *I* ^2^ test. A random‐effects model was used for the NMA due to the expected significant heterogeneity across trials.

Local and global methods were applied to evaluate consistency, with node splitting used to assess inconsistency (assessed only for loops in the evidence network) [22]. Prediction intervals were also estimated to assess the extent to which common heterogeneity affects the relative effect with respect to uncertainty in future studies. Summary RRs and MDs for all pairwise comparisons were presented in a league table. Treatments were ranked using the surface under the cumulative ranking (SUCRA) curve and mean ranks. A network graph was used to illustrate relationships between interventions and specific outcomes. Funnel plots were used to detect potential publication bias [23]. The Stata software tool (Version 15.1) was used for all analyses.

### 2.8. Declaration of AI Tool Usage

No Artificial Intelligence Generated Content (AIGC) tools were used in the preparation of this manuscript.

## 3. Results

### 3.1. Study Selection

**FIGURE 1:**
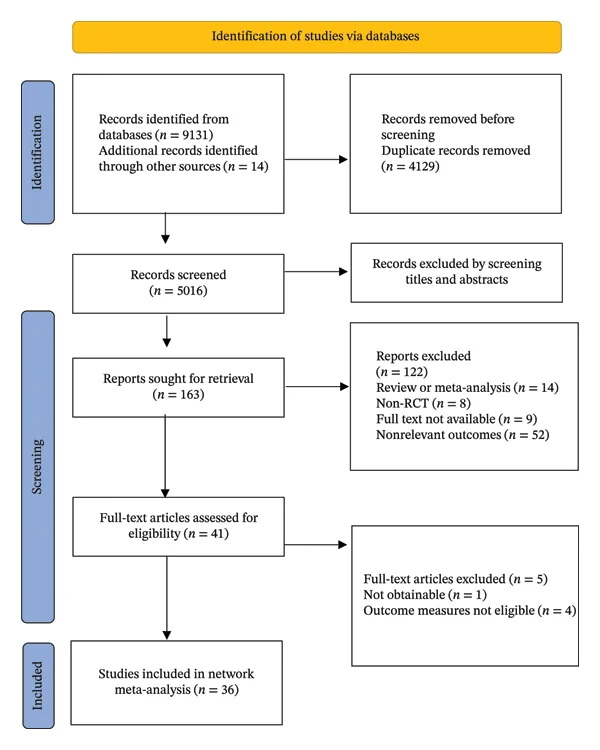
PRISMA flow diagram.

Figure 1 illustrates the study selection process. A total of 5016 unique studies were identified, from which 36 RCTs [24–59] involving 3123 participants were included in the NMA. These studies evaluated the efficacy of 20 different interventions for neurogenic cancer pain. One study [24] reported that when patients with cancerous pudendal neuralgia were treated with oxycontin and pregabalin, the addition of celecoxib effectively alleviated their pain and reduced their consumption of oxycodone. Two studies [25, 43] showed that both duloxetine and pregabalin effectively ameliorated neuropathic pain in patients with lung cancer. A Japanese study [26] suggested that adding duloxetine to opioid and pregabalin therapy might have clinical benefits in the management of cancer‐related neuropathic pain. Three of the studies [28, 56, 57] provided evidence that neurotropin can effectively relieve NCP. Three other studies [27, 29, 54] showed that pregabalin can better control NCP. Three more studies [30, 36, 38] demonstrate that gabapentin can effectively improve the analgesic effect of opioid treatment in patients with NCP, although these results were disputed in another study [51]. Other studies compared the effect of placebo treatment with that of a spicamycin derivative (SD) [31], tramadol [37], topical application of a *Costus* sp. ointment [44], nabiximols [46], LC07 (an external compound Chinese herbal extract including *Herba Geranii*) [49], and *Crocus sativus* L. [59] and found significant amelioration of NCP. In contrast, limited effects were found in studies that compared placebo treatment with amitriptyline [39] and with ketamine in combination with amitriptyline [47], lamotrigine [50], venlafaxine [52], and nortriptyline [53]. The effect of ketamine was found to be comparable with that of placebo in the treatment of NCP [32], although these results were inconsistent with those from another study [40]. Amitriptyline was shown to be more cost‐effective and associated with higher treatment compliance compared to gabapentin [33]. A four‐arm study [34] demonstrated that the efficacies of amitriptyline, gabapentin, and pregabalin were significantly higher than those of the placebo, with the highest efficacy corresponding to pregabalin; therefore, this treatment could be used to reduce the dosage of morphine. Naproxen showed a slightly better therapeutic effect on NCP compared with sustained‐release morphine tablets [41]. Duloxetine was found in two studies to be beneficial to treat chemotherapy‐induced peripheral neuropathy [45, 48]. The analgesic effect of Taylorine tablets in neuropathic pain was equivalent to that provided by morphine alone [55]. When compared with placebo treatment, short‐term use of lidocaine was ineffective in ameliorating NCP according to one study [42], although these results were disputed in another one [58]. Finally, low‐dose gabapentin combined with antidepressants and opioids was reported to be effective in the treatment of NCP, without serious adverse reactions [35].

**FIGURE 2:**
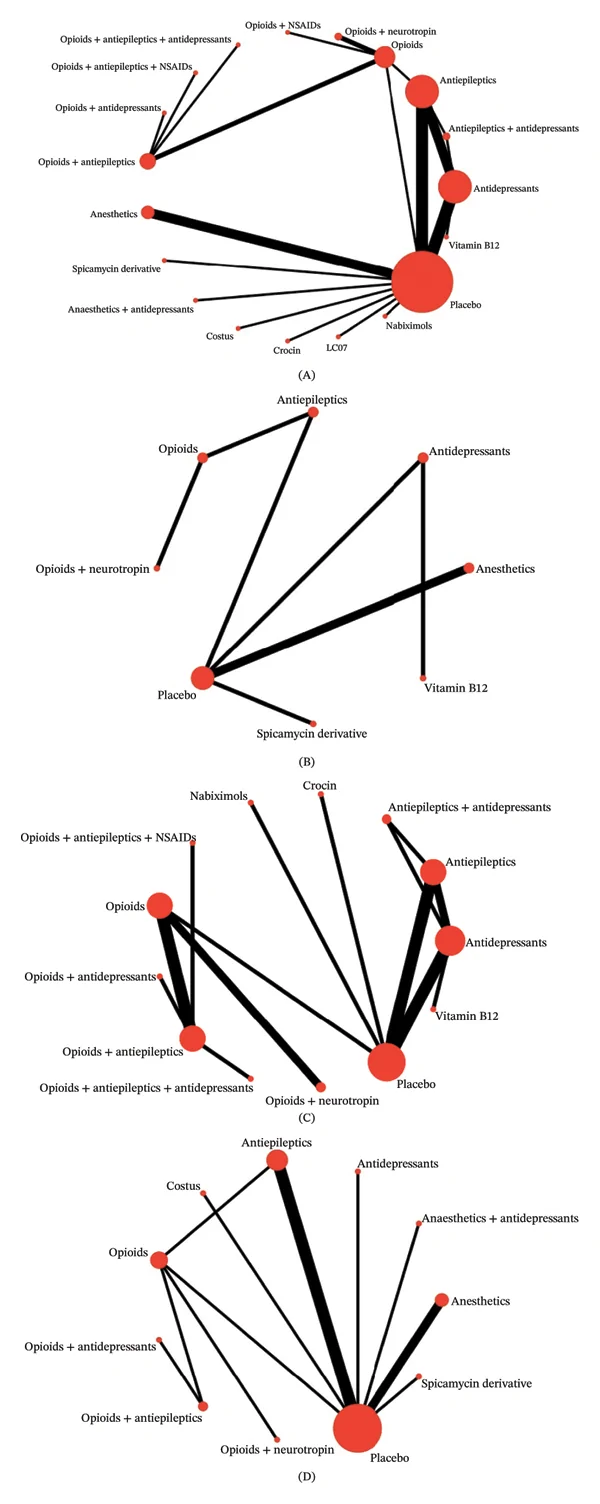
Network meta‐analysis graphs of all outcomes. (A) Pain intensity. (B) Pain relief. (C) Nausea and vomiting. (D) Overall adverse events. The red nodes represent the interventions being compared, while the edges represent the direct comparisons available between pairs of interventions. Line width is proportional to the number of randomized controlled trials comparing each pair of treatments, and the size of every node is proportional to the number of randomized participants (sample size).

The characteristics of the RCTs included in the analysis are detailed in Supporting Table S3. Twenty unique treatments were analyzed, with the abbreviations for medication classes and their respective treatments listed in Table 1. Network plots for eligible comparisons across all outcomes are shown in Figure 2. Placebo and opioids were the most frequently studied interventions, followed by antidepressants and antiepileptics.

**TABLE 1:** List of medication classes and individual treatments.

Class (abbreviations)	Treatments
Anesthetics	Ketamine
	Lidocaine

Antidepressants	Duloxetine
	Imipramine
	Amitriptyline
	Venlafaxine
	Nortriptyline

Antiepileptics	Pregabalin
	Gabapentin
	Lamotrigine

Opioids	Morphine
	Fentanyl
	Oxycodone
	Opioid
	MS Contin
	Methadone
	Tramadol

Nonsteroidal anti‐inflammatory drugs (NSAIDs)	Naproxen
	Celecoxib

Placebo	Placebo

*Combination therapies*	
Antiepileptics + antidepressants (AA) Opioids + NSAIDs (OAce)	Gabapentin + imipramine oxycodone‐acetaminophen
Opioids + antidepressants (OAntidep)	Amityrptiline + tramadol
Opioids + antiepileptics (OAntiepi)	Fentanyl + pregabalin
	OxyContin + pregabalin
	Opioid + pregabalin
	Morphine + pregabalin
	Gabapentin + tramadol
	Gabapentin + opioid
Opioids + antiepileptics + NSAIDs (OAN)	OxyContin + pregabalin + celecoxib
Opioids + neurotropin (ON)	Neurotropin + oxycodone
Opioids + antiepileptics + antidepressants (OAA)	Opioid + pregabalin + duloxetine
Anesthetics + antidepressants (KA)	Ketamine + amitriptyline

*Others*	
Costus	Costus
Crocin	Crocus
LC07	LC07
Nabiximols	Nabiximols
Spicamycin Derivative (SD)	Spicamycin Derivative
Vitamin B12(VB12)	VB12

### 3.2. Risk of Bias

**FIGURE 3:**
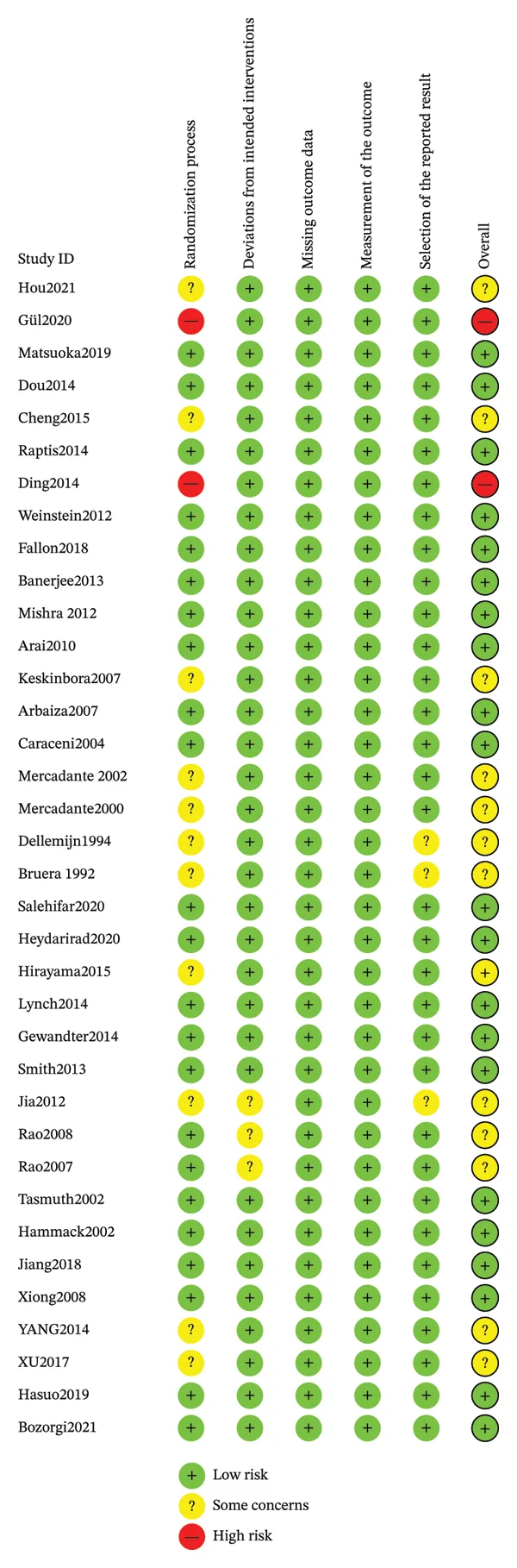
Cochrane risk of bias graph.

The RoB 2.0 tool, designed to evaluate the risk of bias in randomized trials, was used to assess the quality of the RCTs across five domain criteria. Of the 36 studies, 21 (58.3%) had a low risk of bias, 13 (36.1%) had some concerns, and 2 (5.6%) had a high risk of bias. Most trials with uncertain risk did not report random assignment concealment (Figure 3).

### 3.3. Assessment of Clinical Hypotheses

The mean age of the participants across the included trials ranged from 49.0 to 68.4 years, with a median age of 59 years. The proportion of male participants ranged from 0% to 70%, with a median of 50%. All patients had a diagnosis of cancer‐related pain, neuralgia, or neuralgia secondary to radiotherapy or chemotherapy. The distributions of age and sex were comparable across the study populations (Supporting Table S3), supporting the transitivity hypothesis for the dataset.

### 3.4. Primary Outcome

#### 3.4.1. Pain Intensity

The NMA demonstrated that the combination of opioids with antiepileptics and nonsteroidal anti‐inflammatory drugs (OAN) (weighted mean difference [WMD], −3.49; 95% CI, −6.08 to −0.89), antiepileptics with antidepressants (AA; WMD, −3.32; 95% CI, −5.02 to −1.62), LC07 (WMD, −2.18; 95% CI, −4.06 to −0.30), SD (WMD, −2.00; 95% CI, −3.97 to −0.03), anesthetics (WMD, −1.54; 95% CI, −2.71 to −0.36), and antiepileptics (WMD, −0.80; 95% CI, −1.52 to −0.08) significantly reduced pain intensity compared with placebo. In contrast, crocin, nabiximols, opioids, antidepressants, vitamin B12, *Costus* sp. ointment, and the combinations of opioids with antiepileptics and antidepressants (OAA), opioids with neurotropin (ON), opioids with antidepressants (OAntidep), opioids with antiepileptics (OAntiepi), and anesthetics with antidepressants (KA) failed to significantly reduce pain intensity compared with the placebo.

**FIGURE 4:**
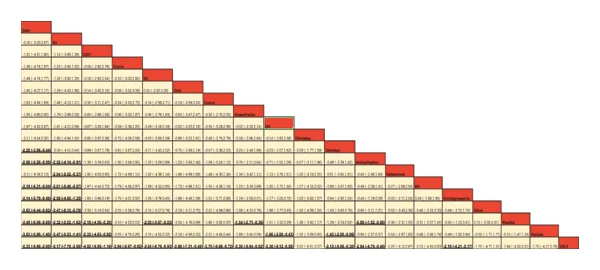
Network meta‐analysis results of pain intensity. Treatments are ranked in descending order of surface under the curve cumulative ranking (SUCRA) for pain intensity. The league table should be read from left to right; the estimate in each cell represents the effect of the column‐defining treatment compared to the row‐defining treatment. For continuous outcomes, such as pain intensity, effects are reported as mean differences (MDs). In the left half of the table, an MD less than 0 favors the column‐defining treatment. Statistically significant results are highlighted in bold. Abbreviations: AA, antiepileptics with antidepressants; OAN, opioids with antiepileptics and nonsteroidal anti‐inflammatory drugs (NSAIDs); SD, spicamycin derivative; OAA, opioids with antiepileptics and antidepressants; ON; opioids with neurotropin; OAntidep, opioids with antidepressants; OAntiepi, opioids with antiepileptics; KA, anesthetics with antidepressants.

League tables (Figure 4) indicated that triple combinations of opioids with other drugs were more effective than combinations with a single drug or opioids alone, while opioids and vitamin B12 were no more effective than placebo in reducing pain intensity. According to SUCRA rankings, OAN had the highest probability of being the most effective treatment to reduce pain intensity (0.91), followed by AA (0.90) and LC07 (0.72) (Supporting Table S4A).

### 3.5. Secondary Outcomes

#### 3.5.1. Pain Relief Rate

The league table for pain relief rate (Supporting Information Figure S1A) showed no significant differences for any pairwise interventions. SUCRA rankings indicated that ON (0.81) and antiepileptics (0.76) were the treatments most likely to be effective, while vitamin B12 (0.15) was the least likely (Supporting Table S4B).

#### 3.5.2. Nausea and Vomiting

Compared with OAA, most other interventions were associated with significantly lower risks of nausea and vomiting. The detailed RR estimates for each comparison are presented in Supporting Figure S1B. According to SUCRA rankings, vitamin B12 (0.90) was most likely to be the safest treatment, followed by antiepileptics (0.87) and placebo (0.72) (Supporting Table S4C).

#### 3.5.3. Overall Adverse Events

Compared with opioids, antidepressants (RR, 0.03; 95% CI, 0.12–0.75), ketamine with amitriptyline (RR, 0.38; 95% CI, 0.17–0.85), placebo (RR, 0.40; 95% CI, 0.20–0.80), and antiepileptics (RR, 0.43; 95% CI, 0.24–0.79) were associated with significantly lower risks of overall adverse events (Supporting Figure S1C). Opioids, their combinations, and SD exhibited a higher overall incidence of adverse events. SUCRA rankings indicated that antidepressants (0.86) were most likely to be the safest treatment, followed by ketamine with amitriptyline (0.73) and placebo (0.70), while opioids with neurotropin (0.10) were associated with the highest probability of adverse events (Supporting Table S4D).

### 3.6. Evaluation of Statistical Inconsistency

Global inconsistency testing revealed no significant inconsistencies for any of the outcomes (Supporting Table S5). No indirect comparisons were available for pain relief rates; thus, no global inconsistency tests were conducted for them. Similarly, side‐splitting did not identify statistical inconsistencies for any outcome (Supporting Figure S2A‐S2D).

### 3.7. Small‐Study Effects

Comparison‐adjusted funnel plots indicated evidence of publication bias or small‐study effects for all outcomes: pain intensity, pain relief rate, nausea and vomiting, and total adverse events (Supporting Figure S3A‐S3D).

### 3.8. Quality of Evidence Assessment

All outcomes were assessed using the CINeMA approach for indirect and mixed comparisons. Overall, the certainty of evidence for the four primary and secondary outcomes was low (Supporting Table S6A). For the primary outcome, 84.8% of the comparisons were rated as being of low or very low confidence (Supporting Table S6A). The evidence for nausea and vomiting and the other outcomes was graded as lower quality in the CINeMA assessment (Supporting Tables S6B‐S6D).

## 4. Discussion

This systematic review and NMA of treatments for NCP included data from 36 clinical trials, including 3123 patients randomized to 20 distinct treatment protocols or placebo groups. Most studies (34 out of 36; [94.4%]) demonstrated low or unclear risks of bias. For the CINeMA evaluation of pain intensity, we considered the minimally important difference (MID) as the smallest change in patient‐reported outcomes deemed significant by the patient. A value of 1 cm on the 10 cm VAS for pain was used as the MID [60, 61]. Crucially, the CINeMA results indicated that the quality of evidence for each outcome was low or very low; summary treatment effect precision varied considerably, with higher uncertainty observed for certain treatment protocols or those with only a few or no placebo‐controlled trials available. This overall context of low certainty underpins the interpretation of all the findings reported here. When interpreting the clinical relevance of these effect estimates against the MID of 1 cm on the 10 cm VAS, several interventions demonstrated improvements exceeding this threshold. The triple combination OAN (WMD, −3.49; 95% CI, −6.08 to −0.89) and the combination of antiepileptics with antidepressants (WMD, −3.32; 95% CI, −5.02 to −1.62) showed mean pain reductions substantially above the MID, suggesting clinically meaningful analgesic effects. LC07 (WMD, −2.18), SD (WMD, −2.00), and anesthetics (WMD, −1.54) also exceeded the MID, although these estimates were derived from limited evidence. In contrast, antiepileptics alone (WMD, −0.80; 95% CI, −1.52 to −0.08) had a point estimate below the MID, and the CI included values of uncertain clinical importance. However, given the low certainty of most estimates and the wide CIs for several comparisons, all treatment effects reported in this NMA should be interpreted as exploratory rather than definitive.

This is the first study to comprehensively synthesize evidence on the safety and efficacy of 20 distinct therapeutic interventions for NCP. Our synthesis of the comparative evidence suggests that antiepileptic drugs and antidepressants, alone or in combination, can reduce NCP, consistent with the findings of a previous study [9]. We found no significant difference in pain relief between opioids combined with antidepressants or opioids alone, as previously reported [8]. However, consistent with previous results [27, 36], OAntiepi were more effective than opioids alone, supporting the clinical use of this combination for managing NCP. Exploratory findings from the network suggest that ON and OAN were also more effective than opioids alone, potentially due to variations in the molecular mechanisms of chemotherapy‐induced neuropathy [62, 63]. Consequently, neuropathy caused by different chemotherapy agents may respond differently to different drugs, and combining different drug classes may provide better therapeutic coverage.

Based on single‐trial evidence, some plant‐derived drugs such as crocin and LC07 demonstrated better efficacy and fewer adverse effects compared with the placebo, although this requires confirmation. Saffron (*C. sativus* L.), a traditional Persian medicine, contains active compounds with analgesic and neuroprotective properties; some studies suggest that the analgesic effects of crocin may involve an endocannabinoid mechanism [64]. LC07, a Chinese herbal extract containing *Herba Geranii*, may reduce the release of pain‐related neurotransmitters and promote nerve growth factor release and intraepidermal nerve fiber regeneration [49]. Among the interventions evaluated, the comparative efficacy of antiepileptics, antidepressants, and their combinations is supported by multiple trials, whereas findings for plant‐derived compounds (e.g., crocin, LC07, and *Costus* sp.) are derived from single studies and should therefore be considered hypothesis‐generating.

In contrast, opioids or vitamin B12 alone did not demonstrate significantly greater efficacy than placebo, with no significant differences in adverse events between vitamin B12 and placebo treatment. Moreover, opioid combinations did not demonstrate significantly greater efficacy than placebo, except for the particular case of OAN, and they increased the risk of adverse events. However, this finding should be interpreted with caution. The included studies exhibited considerable heterogeneity in patient populations (e.g., varying etiologies of neuropathic pain), and several comparisons involving opioids were supported by limited sample sizes, resulting in wide CIs and reduced statistical power. Moreover, the overall certainty of evidence for the primary outcome was low. Therefore, the present network estimates should not be interpreted as general evidence that opioids are ineffective for NCP; rather, they highlight the need for better‐powered, more homogeneous trials to define the precise role of opioids in this setting. A more consistent finding across studies is that antiepileptic drugs and antidepressants exhibited relatively good efficacy and safety profiles.

Regarding safety, several interventions were associated with a significantly lower risk of nausea and vomiting compared to OAA. These included vitamin B12 (RR, 0.02; 95% CL, 0.00–0.56), antiepileptics (RR, 0.05; 95% CL, 0.01–0.32), placebo (RR, 0.09; 95% CL, 0.00–0.47), antidepressants (RR, 0.12; 95% CL, 0.02–0.80), and the combination of opioids with antidepressants (RR, 0.18; 95% CL, 0.03–0.99), opioids with neurotropin (RR, 0.18; 95% CL, 0.04–0.85), OAntiepi (RR, 0.21; 95% CL, 0.07–0.65), and OAN (RR, 0.21; 95% CL, 0.05–0.87). The SUCRA rankings corroborated these findings, indicating that vitamin B12 was associated with the highest probability of being the safest treatment for nausea and vomiting (90.2%), followed by antiepileptics (87.1%) and placebo (72.3%). This suggests that vitamin B12 and antiepileptics simply lack the emetogenic (nausea‐inducing) potential of opioids, while the effect of the placebo may reflect the influence of psychological factors on adverse events. For overall adverse events, antidepressants (RR, 0.03; 95% CL, 0.12–0.75), ketamine with amitriptyline (RR, 0.38; 95% CL, 0.17–0.85), placebo (RR, 0.40; 95% CL, 0.20–0.80), and antiepileptics (RR, 0.43; 95% CL, 0.24–0.79) were associated with a significantly lower risk compared to opioids alone. In contrast, opioids, their combinations, and SD were associated with a higher number of overall adverse events. According to the SUCRA rankings, antidepressants were associated with the highest probability of being the safest treatment (85.7%), followed by ketamine and amitriptyline (73.2%) and placebo (69.8%), whereas ON was associated with the highest probability of adverse events (10.2%). Adverse events, particularly with opioids or their combinations, occurred more frequently than with placebo or non‐opioid treatments. These safety profiles provide clinically relevant insights, suggesting that opioids may not confer greater benefits than less harmful interventions and that there is considerable variability in the efficacy of the available treatment options for NCP. Contrary to the belief in the superiority of opioids for cancer‐related pain, our evidence suggests that certain non‐opioid drugs may be more effective for the treatment of NCP. Further assessment of potential reporting biases, as suggested by the observed asymmetry in the funnel plot for overall adverse events (Figure S3D), warrants consideration. This asymmetry likely stems from a combination of factors, including the predominance of small‐sample studies in the analysis, the variable methodological quality of the included studies, and the clinical heterogeneity of the study population (specifically the mixed etiologies of the neuropathic pain). Therefore, the funnel plot asymmetry suggests caution in the interpretation of the exact magnitude of effect sizes.

Overall, the network comparisons indicate that triple combinations of opioids with other agents were more effective than double combinations or opioids alone, but the latter showed limited efficacy and were significantly associated with adverse events. Antiepileptics and antidepressants exhibited better efficacy and fewer adverse reactions. Although vitamin B12 was safe, its efficacy was limited. Despite these observations, the low certainty of the underlying evidence warrants caution in interpreting the comparative effectiveness of these regimens. The SUCRA rankings and effect estimates should not be interpreted as firm hierarchies of treatment efficacy, but rather as preliminary signals that require corroboration in well‐designed, adequately powered trials. The exploratory nature of these findings is particularly relevant for interventions supported by only a single trial.

### 4.1. Limitations

This study has certain limitations that should be considered when interpreting the results. First, the evidence for certain plant‐derived therapeutic agents, such as crocin, LC07, and *Costus* sp., was limited to a single trial, increasing the risk of overestimating their treatment effects. Therefore, although promising, the results should be considered exploratory and hypothesis‐generating. Second, only 58.3% of the included RCTs had a low risk of bias, and CINeMA assessments demonstrated imprecision, leading to low or very low certainty for most pharmaceutical interventions. Third, the trial populations consisted of patients with neuropathic pain of mixed etiology, including chemotherapy‐induced pain and tumor infiltration. Consequently, the findings are derived from a heterogeneous cohort, and their generalizability to specific subpopulations may be limited. Moreover, the limited number of studies precluded subgroup or meta‐regression analyses by patient subpopulation, pain type, or time point. Additionally, some agents, such as imipramine, amitriptyline, and venlafaxine, were administered at doses lower than those clinically recommended [25, 39, 52], which may have restricted their efficacy, influencing the overall results. The effect estimates for SD and for the combination of ketamine and amitriptyline [31, 47] should also be interpreted cautiously. Therefore, these findings warrant careful consideration. High‐quality, well‐designed, rigorous RCTs are needed to collect more robust evidence regarding the efficacy and safety of pharmacological treatments for NCP.

## 5. Conclusions

In summary, based on the evidence currently available, antiepileptics, antidepressants (alone or in combination), and some opioid combinations are most likely to be effective in the management of NCP. Although some plant‐derived compounds also demonstrated potential analgesic effects, the evidence is based on a limited number of trials, and the findings should be considered exploratory. Additional evidence is needed to verify their efficacy. Clinicians should perform individualized assessments, balancing potential benefits with the risk of treatment‐related adverse reactions to optimize pain relief and minimize side effects in patients with NCP.

## Author Contributions

Ping Huang and Tingting Nan developed the review protocol. Ping Huang and Ruping Ni conducted the literature searches, extracted data, and assessed the methodological quality of the included studies. Ping Huang and Yuhong Huang performed the network meta‐analyses. Ping Huang drafted the manuscript. Tingting Nan, Maobai Liu, and Jing Yang interpreted the results and edited the manuscript. Jing Yang obtained the funding to support this work. All authors discussed the results.

## Funding

This work was supported by the Fujian Medical University Undergraduate Innovation and Entrepreneurship Teaching Reform Project (Grant number FBJG20210119); the Fujian Provincial Natural Science Foundation of China (Grant number 2024J011600); and the Startup Fund for scientific research, Fujian Medical University (Grant number 2023QH1258).

## Disclosure

All authors approved the final manuscript for publication.

## Ethics Statement

The authors have nothing to report.

## Consent

The authors have nothing to report.

## Conflicts of Interest

The authors declare no conflicts of interest.

## Supporting Information

Additional supporting information can be found online in the Supporting Information section.

## Supporting information


**Supporting Information** Table S1, PRISMA NMA Checklist. Table S2, Search Strategy. Table S3, Characteristics of the studies included in the network meta‐analysis. Table S4, Probability rank and SUCRA for all outcomes. Figure S1A, Network meta‐analysis results for pain relief. Figure S1B, Network meta‐analysis results for nausea and vomiting. Figure S1C, Network meta‐analysis results for overall adverse events. Table S5, Global inconsistency. Figure S2, Node inconsistency. Figure S3, Funnel plots for all outcomes. Table S6, Grading of the evidence used in the network meta‐analysis through CINeMA.

## Data Availability

The data that support the findings of this study are available in the supporting information of this article.
