# Supplementary material for: Adjusted Indirect and Mixed Comparisons of Drug Therapy for Neuropathic Cancer Pain
Source: Pain Res Manag. 2026 Aug 30;2026:9970065. doi: 10.1155/prm/9970065 (PMC13527231; doi:10.1155/prm/9970065)
Supplement: Supplementary file 1 [file PRM-2026-9970065-s001.zip › Supplementary Material_1.docx]

**Supplementary Online Content**

**Table S1 PRISMA NMA Checklist**

**Table S2 Search Strategy**

**Table S3 Characteristics of the studies included in the network meta-analysis**

**Table S4 Probability rank and SUCRA of All Outcomes**

**Figure S1A network meta-analysis results of pain relief**

**Figure S1B network meta-analysis results of nausea and vomiting**

**Figure S1C network meta-analysis results of overall adverse events**

**Table S5 Global inconsistency**

**Figure S2 Node inconsistency**

**Figure S3 Funnel Plots of All Outcomes**

**Table S6 Grading the evidence of the network meta-analysis using CINeMA**

**Table S1 PRISMA NMA Checklist**

| **Section/Topic** | **Item** | **Checklist Item** | **Reported on Page** |
| --- | --- | --- | --- |
| **TITLE** |  |  |  |
| Title | 1 | Identify the report as a systematic review *incorporating a network meta-analysis (or related form of meta-analysis).* | 1 |
| **ABSTRACT** |  |  |  |
| Structured summary | 2 | Provide a structured summary including, as applicable:  **Background:** main objectives  **Methods:** data sources; study eligibility criteria, participants, and interventions; study appraisal; and *synthesis methods, such as network meta-analysis.*  **Results:** number of studies and participants identified; summary estimates with corresponding confidence/credible intervals; *treatment rankings may also be discussed. Authors may choose to summarize pairwise comparisons against a chosen treatment included in their analyses for brevity.*  **Discussion/Conclusions:** limitations; conclusions and implications of findings.  **Other:** primary source of funding; systematic review registration number with registry name. | 1-2 |
| **INTRODUCTION** |  |  |  |
| Rationale | 3 | Describe the rationale for the review in the context of what is already known*, including mention of why a network meta-analysis has been conducted.* | 3-4 |
| Objectives | 4 | Provide an explicit statement of questions being addressed, with reference to participants, interventions, comparisons, outcomes, and study design (PICOS). | 3-4 |
| **METHODS** |  |  |  |
| Protocol and registration | 5 | Indicate whether a review protocol exists and if and where it can be accessed (e.g., Web address); and, if available, provide registration information, including registration number. | 5 |
| Eligibility criteria | 6 | Specify study characteristics (e.g., PICOS, length of follow-up) and report characteristics (e.g., years considered, language, publication status) used as criteria for eligibility, giving rationale. *Clearly describe eligible treatments included in the treatment network, and note whether any have been clustered or merged into the same node (with justification).* | 5 |
| Information sources | 7 | Describe all information sources (e.g., databases with dates of coverage, contact with study authors to identify additional studies) in the search and date last searched. | 5 |
| Search | 8 | Present full electronic search strategy for at least one database, including any limits used, such that it could be repeated. | 5, Table S2 |
| Study selection | 9 | State the process for selecting studies (i.e., screening, eligibility, included in systematic review, and, if applicable, included in the meta-analysis). | 5，Figure 1 |
| Data collection process | 10 | Describe method of data extraction from reports (e.g., piloted forms, independently, in duplicate) and any processes for obtaining and confirming data from investigators. | 5-6 |
| Data items | 11 | List and define all variables for which data were sought (e.g., PICOS, funding sources) and any assumptions and simplifications made. | 6 |
| **Geometry of the network** | **S1** | Describe methods used to explore the geometry of the treatment network under study and potential biases related to it. This should include how the evidence base has been graphically summarized for presentation, and what characteristics were compiled and used to describe the evidence base to readers. | 10 |
| Risk of bias within individual studies | 12 | Describe methods used for assessing risk of bias of individual studies (including specification of whether this was done at the study or outcome level), and how this information is to be used in any data synthesis. | 6 |
| Summary measures | 13 | State the principal summary measures (e.g., risk ratio, difference in means). *Also describe the use of additional summary measures assessed, such as treatment rankings and surface under the cumulative ranking curve (SUCRA) values, as well as modified approaches used to present summary findings from meta-analyses.* | 8-9 |
| Planned methods of analysis | 14 | Describe the methods of handling data and combining results of studies for each network meta-analysis. This should include, but not be limited to:   - *Handling of multi-arm trials;* - *Selection of variance structure;* - *Selection of prior distributions in Bayesian analyses; and* - *Assessment of model fit.* | 8-9 |
| **Assessment of Inconsistency** | **S2** | Describe the statistical methods used to evaluate the agreement of direct and indirect evidence in the treatment network(s) studied. Describe efforts taken to address its presence when found. | 8-9 |
| Risk of bias across studies | 15 | Specify any assessment of risk of bias that may affect the cumulative evidence (e.g., publication bias, selective reporting within studies). | 8-9 |
| Additional analyses | 16 | Describe methods of additional analyses if done, indicating which were pre-specified. This may include, but not be limited to, the following:   - Sensitivity or subgroup analyses; - Meta-regression analyses; - *Alternative formulations of the treatment network; and* - *Use of alternative prior distributions for Bayesian analyses (if applicable).* | 6-7 |
| **RESULTS** |  |  |  |
| Study selection | 17 | Give numbers of studies screened, assessed for eligibility, and included in the review, with reasons for exclusions at each stage, ideally with a flow diagram. | 10-11, Figure 1 |
| **Presentation of network structure** | **S3** | Provide a network graph of the included studies to enable visualization of the geometry of the treatment network. | 10，Figure 2 |
| **Summary of network geometry** | **S4** | Provide a brief overview of characteristics of the treatment network. This may include commentary on the abundance of trials and randomized patients for the different interventions and pairwise comparisons in the network, gaps of evidence in the treatment network, and potential biases reflected by the network structure. | 10-11 |
| Study characteristics | 18 | For each study, present characteristics for which data were extracted (e.g., study size, PICOS, follow-up period) and provide the citations. | 11, Table S3 |
| Risk of bias within studies | 19 | Present data on risk of bias of each study and, if available, any outcome level assessment. | 10-11, Figure 3 |
| Results of individual studies | 20 | For all outcomes considered (benefits or harms), present, for each study: 1) simple summary data for each intervention group, and 2) effect estimates and confidence intervals. *Modified approaches may be needed to deal with information from larger networks.* | 11-12 |
| Synthesis of results | 21 | Present results of each meta-analysis done, including confidence/credible intervals. *In larger networks, authors may focus on comparisons versus a particular comparator (e.g. placebo or standard care), with full findings presented in an appendix. League tables and forest plots may be considered to summarize pairwise comparisons.* If additional summary measures were explored (such as treatment rankings), these should also be presented. | 11-14,  Figure 4  Table S4A-4D,  Figure S1A,  Figure S1B,  Figure S1C |
| **Exploration for inconsistency** | **S5** | Describe results from investigations of inconsistency. This may include such information as measures of model fit to compare consistency and inconsistency models, *P* values from statistical tests, or summary of inconsistency estimates from different parts of the treatment network. | 13,  Figure S2,  Table S5 |
| Risk of bias across studies | 22 | Present results of any assessment of risk of bias across studies for the evidence base being studied. | 13,  Figure S3 |
| Results of additional analyses | 23 | Give results of additional analyses, if done (e.g., sensitivity or subgroup analyses, meta-regression analyses*, alternative network geometries studied, alternative choice of prior distributions for Bayesian analyses,* and so forth). | Table S6A-6D |
| **DISCUSSION** |  |  |  |
| Summary of evidence | 24 | Summarize the main findings, including the strength of evidence for each main outcome; consider their relevance to key groups (e.g., healthcare providers, users, and policy-makers). | 15-18 |
| Limitations | 25 | Discuss limitations at study and outcome level (e.g., risk of bias), and at review level (e.g., incomplete retrieval of identified research, reporting bias). *Comment on the validity of the assumptions, such as transitivity and consistency. Comment on any concerns regarding network geometry (e.g., avoidance of certain comparisons).* | 17-18 |
| Conclusions | 26 | Provide a general interpretation of the results in the context of other evidence, and implications for future research. | 18 |
| **FUNDING** |  |  |  |
| Funding | 27 | Describe sources of funding for the systematic review and other support (e.g., supply of data); role of funders for the systematic review. This should also include information regarding whether funding has been received from manufacturers of treatments in the network and/or whether some of the authors are content experts with professional conflicts of interest that could affect use of treatments in the network. | 18 |

Table S2 **Search Strategy**

Search strategy in PubMed.

| No. | Query | Results |
| --- | --- | --- |
| 7 | ((("Neuralgia"[Mesh]) OR (neuralgia* OR neurodynia* OR ((nerve OR neuropathic) AND (ache OR pain*)))) AND (("Neoplasms"[Mesh]) OR (cancer* OR neoplas* OR mass OR lesion* OR nodul* OR tumor* OR tumour* OR malignan* OR metastas* OR carcinoma*))) AND ((("Randomized Controlled Trial" [Publication Type]) OR (randomized[Title/Abstract])) OR (placebo[Title/Abstract])) | 1,818 |
| 6 | (("Randomized Controlled Trial" [Publication Type]) OR (randomized[Title/Abstract])) OR (placebo[Title/Abstract]) | 1,066,284 |
| 5 | ("Neoplasms"[Mesh]) OR (cancer* OR neoplas* OR mass OR lesion* OR nodul* OR tumor* OR tumour* OR malignan* OR metastas* OR carcinoma*) | 7,919,273 |
| 4 | "Neoplasms"[Mesh] | 3,980,203 |
| 3 | ("Neuralgia"[Mesh]) OR (neuralgia* OR neurodynia* OR ((nerve OR neuropathic) AND (ache OR pain*))) | 124,253 |
| 2 | neuralgia* OR neurodynia* OR ((nerve OR neuropathic) AND (ache OR pain*)) | 126,459 |
| 1 | "Neuralgia"[Mesh] | 25,689 |

Search strategy in EMBASE.

| No. | Query | Results |
| --- | --- | --- |
| #10 | #3 AND #8 AND #9 | 3,231 |
| #9 | #6 OR #7 | 8,867,760 |
| #8 | #4 OR #5 | 216,559 |
| #7 | cancer*:ti,ab,kw OR neoplas*:ti,ab,kw OR mass:ti,ab,kw OR lesion*:ti,ab,kw OR nodul*:ti,ab,kw OR tumor*:ti,ab,kw OR tumour*:ti,ab,kw OR malignan*:ti,ab,kw OR metastas*:ti,ab,kw | 7,808,852 |
| #6 | ‘malignant neoplasm’/exp | 4,749,608 |
| #5 | neuralgia*:ti,ab,kw OR neurodynia*:ti,ab,kw OR ((nerve:ti,ab,kw OR neuropathic:ti,ab,kw) AND (ache:ti,ab,kw OR pain*:ti,ab,kw)) | 133,770 |
| #4 | ‘neuralgia’/exp | 141,019 |
| #3 | #1 OR #2 | 2,312,505 |
| #2 | ‘randomized controlled trial’/exp | 828,863 |
| #1 | ‘randomized controlled trial’:ti,ab,kw OR random*:ti,ab,kw OR placebo:ti,ab,kw | 2,204,428 |

Search strategy in Cochrane database.

| ID | Search | Results |
| --- | --- | --- |
| #1 | MeSH descriptor: [Neuralgia] explode all trees | 2444 |
| #2 | (neuralgia* OR neurodynia* OR ((nerve OR neuropathic) AND (ache OR pain*))):ti,ab,kw | 27228 |
| #3 | MeSH descriptor: [Neoplasms] explode all trees | 125041 |
| #4 | (cancer* OR neoplas* OR mass OR lesion* OR nodul* OR tumor* OR tumour* OR malignan* OR metastas*): ti,ab,kw | 412629 |
| #5 | #1 OR #2 | 27594 |
| #6 | #3 OR #4 | 423884 |
| #7 | #5 AND #6 | 4082 |

**Table S3 Characteristics of the studies included in the network meta-analysis**

| Author，year | Country | Trial type | Age(SD) | Male/n(%) | Control group | Experimental group | Pain Scale（range） | Follow-up | N |
| --- | --- | --- | --- | --- | --- | --- | --- | --- | --- |
| Hou2021 | China | Parallel | 57.87(13.23) | 25(49.02%) | OxyContin+Pregabalin | OxyContin+Pregabalin+celecoxib | NRS(0-10) | 14d | 51 |
| Gül2020 | Turkey | Parallel | 57.88(11.35) | 28(66.67%) | duloxetine | pregabalin | VAS(0-100) | 3M | 44 |
| Matsuoka2019 | Japan | two-parallel group | 62.9(10.6) | 43(61.4%) | Opioid+Pregabalin | Opioid+Pregabalin+duloxetine | BPI-5(0-10) | 10d | 70 |
| Dou2014 | china | crossover | 56.5(12.1) | 24(60%) | morphine | morphine+pregabalin | NRS(0-10) | 5w | 40 |
| Cheng2015 | china | Parallel | NR | NR | oxycodone | Neurotropin+oxycodone | VAS(0-10) | 14d | 102 |
| Raptis2014 | Greece | Parallel | 62.2(10.63) | 58(48.33%) | transdermal fentanyl | pregabalin | VAS(0-10) | 4w | 120 |
| Ding2014 | china | Parallel | 67.1(4.3) | 32(53.3%) | transdermal fentanyl | gabapentin+transdermal fentanyl | VAS(0-10) | 4w | 60 |
| Weinstein2012 | America | Parallel | 61.7(12.2) | 10(52.6%) | placebo | Spicamycin Derivative (KRN5500) | NRS(0-10) | 14w | 19 |
| Fallon2018 | United Kingdom | Parallel | 58(11.1) | 73(34.1%) | placebo | Ketamine | NCCN Distress Thermometer(0-10) | 30d | 214 |
| Banerjee2013 | India | Parallel | 53.58(10.23) | 41(53.94%) | amityrptiline+tramadol | gabapentin+tramadol | VAS(0-10) | 6M | 76 |
| Mishra2012 | India | Parallel | NR | NR | placebo | amitriptyline; gabapentin; pregabalin | VAS(0-10) | 4W | 120 |
| Arai2010 | Japan | Parallel | 66.59(9.17) | 34(65.38%) | imipramine | gabapentin+imipramine; gabapentin; gabapentin | NRS(0-10) | 7d | 52 |
| Keskinbora2007 | Turkey | Parallel | 54.87(15.67) | 42(66.67%) | opioid | gabapentin+opioid | NRS(0-10) | 13d | 63 |
| Arbaiza2007 | Peru | Parallel | 49.86(NR) | 14(38.89%) | placebo | Tramadol | NRS(0-10) | 45d | 36 |
| Caraceni2004 | Italy | Parallel | 59.57(10.98) | 53/121(43.8%) | placebo | Gabapentin | NRS(0-10) | 10d | 120 |
| Mercadante2002 | Italy | crossover | 67.13(7.55) | 10/16(62.5%) | placebo | amitriptyline | NRS(0-10) | 2W | 16 |
| Mercadante2000 | Italy | crossover | 57.6(13.95) | 7/10(70%) | placebo | Ketamine | NRS(0-10) | 3d | 10 |
| Dellemijn1994 | The Netherlands. | crossover | 60.1(11.69) | 10/20(50%) | MS Contin | Naproxen | NRS(0-100) | 2W | 20 |
| Bruera1992 | Canada | crossover | 54(17) | 7/11(63.63%) | placebo | Lidocaine | VAS(0-100) | 48h | 11 |
| Salehifar2020 | Iran | Parallel | 49.04(9.59) | 0 | duloxetine | pregabalin | VAS(0-10) | 6W | 82 |
| Heydarirad2020 | Iran | Parallel | 54.5(10.14) | 10/30(33.3%) | placebo | Costus sp.oil | VAS(0-10) | 4W | 30 |
| Hirayama2015 | Japan | crossover | 62.5(6.7) | 17/34(50%) | vitamin B12 | duloxetine | VAS(0-10) | 4W | 34 |
| Lynch2014 | Canada | crossover | 56(10.8) | 3/18(16.67%) | placebo | nabiximols | NRS(0-10) | 4W | 16 |
| Gewandter2014 | America | Parallel | NR | 135/462(29.22%) | placebo | ketamine+amitriptyline | NRS(0-10) | 6W | 458 |
| Smith2013 | America | crossover | 59 (10.5) | 82/220 (37%) | placebo | duloxetine | (BPI-SF)(0-10) | 5W | 220 |
| Jia2012 | China | Parallel | 58(10.75) | 62/102(60.78%) | placebo | LC07 | NRS(0-10) | 7d | 102 |
| Rao2008 | America | Parallel | 61(13.75) | 51/125 (41%) | placebo | lamotrigine | NRS(0-10) | 10W | 125 |
| Rao2007 | America | crossover | 59(14.75) | 31/115 (27%) | placebo | gabapentin | NRS(0-10) | 6W | 115 |
| Tasmuth2002 | Finland | crossover | 55(8.75) | 0 | placebo | venlafaxine | VAS(0-100) | 4W | 15 |
| Hammack2002 | America | crossover | 59.53 | NR | placebo | nortriptyline | VAS(0-100) | 4W | 57 |
| Jiang2018 | China | Parallel | 56.15(8.39) | 77/128(60.16%) | placebo | pregabalin | NRS(0-10) | 16W | 128 |
| Xiong2008 | China | Parallel | 53.85(12.69) | 26/53(49.05%) | morphine | oxycodone-acetaminophen | NRS(0-10) | 7d | 53 |
| YANG2014 | China | Parallel | NR | 65/131(49.62%) | oxycodone | neurotropin+oxycodone | VAS(0-10) | 14d | 156 |
| XU2017 | China | Parallel | 66.12 (1.74) | 59/86(68.6%) | oxycodone | Neurotropin+oxycodone | VAS(0-10) | 14d | 86 |
| Hasuo2019 | Japan | crossover | 68.4 (10.8) | 14/25 (56%) | placebo | lidocaine | NRS(0-10) | 24h | 25 |
| Bozorgi2021 | Iran | crossover | 61.5 (3.6) | 83 (46.8%) | placebo | Crocus sativus L | NRS(0-10) | 8W | 177 |

**Table S4 Probability rank and SUCRA of All Outcomes**

**Table S4A Probability rank and SUCRA of pain intensity**

| Treatment | SUCRA | PrBest | MeanRank |
| --- | --- | --- | --- |
| Opioids+Antiepileptics+NSAIDs | 90.7 | 42.3 | 2.7 |
| Antiepileptics+Antidepressants | 90.4 | 30.9 | 2.7 |
| LC07 | 72 | 5.5 | 6 |
| Crocin | 70.4 | 6.3 | 6.3 |
| Spicamycin Derivative | 68.5 | 4.5 | 6.7 |
| Opioids+Antiepileptics+Antidepressants | 68.2 | 4.6 | 6.7 |
| Costus | 65.8 | 4.2 | 7.2 |
| Anaesthetics | 60.5 | 0.4 | 8.1 |
| Opioids+Neurotropin | 59.5 | 0.3 | 8.3 |
| Opioids+Antidepressants | 54.9 | 0.9 | 9.1 |
| Opioids+Antiepileptics | 53.4 | 0 | 9.4 |
| Antiepileptics | 42.6 | 0 | 11.3 |
| Nabiximols | 32.5 | 0.1 | 13.1 |
| Anaesthetics+antidepressants | 30.3 | 0 | 13.5 |
| Antidepressants | 29.3 | 0 | 13.7 |
| Opioids+NSAIDs | 21.2 | 0 | 15.2 |
| Placebo | 19.3 | 0 | 15.5 |
| Opioids | 17.5 | 0 | 15.9 |
| Vitamin B12 | 2.9 | 0 | 18.5 |

**eTable 4B Probability rank and SUCRA of pain relief**

| Treatment | SUCRA | PrBest | MeanRank |
| --- | --- | --- | --- |
| Opioids+Neurotropin | 81.3 | 50.5 | 2.3 |
| Antiepileptics | 75.6 | 16.7 | 2.7 |
| Antidepressants | 63 | 13.9 | 3.6 |
| Spicamycin Derivative | 50.7 | 14.5 | 4.4 |
| Anaesthetics | 46.9 | 3.3 | 4.7 |
| Opioids | 37.9 | 0.2 | 5.3 |
| Placebo | 29.6 | 0 | 5.9 |
| Vitamin B12 | 15 | 0.9 | 7 |

**Table S4C Probability rank and SUCRA of nausea and vomiting**

| Treatment | SUCRA | PrBest | MeanRank |
| --- | --- | --- | --- |
| Vitamin B12 | 90.2 | 65.9 | 2.2 |
| Antiepileptics | 87.1 | 13.8 | 2.5 |
| Placebo | 72.3 | 0.4 | 4.3 |
| Antiepileptics+Antidepressants | 69.3 | 15.1 | 4.7 |
| Antidepressants | 59.3 | 0.2 | 5.9 |
| Opioids+Antidepressants | 47.6 | 2 | 7.3 |
| Crocin | 47.3 | 1.7 | 7.3 |
| Opioids+Neurotropin | 46.7 | 0.1 | 7.4 |
| Opioids+Antiepileptics | 42.8 | 0 | 7.9 |
| Opioids+Antiepileptics+NSAIDs | 42.5 | 0.5 | 7.9 |
| Opioids | 20.2 | 0 | 10.6 |
| Nabiximols | 19.5 | 0.2 | 10.7 |
| Opioids+Antiepileptics+Antidepressants | 5.1 | 0 | 12.4 |

**Table S4D Probability rank and SUCRA of overall adverse events**

| Treatment | SUCRA | PrBest | MeanRank |
| --- | --- | --- | --- |
| Placebo | 69.8 | 1.4 | 4 |
| Anesthetics | 54.6 | 9.5 | 5.5 |
| Antidepressants | 85.7 | 44.4 | 2.4 |
| Antiepileptics | 60.7 | 1.6 | 4.9 |
| Costus | 23.3 | 9.1 | 8.7 |
| Anesthetics+antidepressants | 73.2 | 12.9 | 3.7 |
| Opioids | 19.4 | 0 | 9.1 |
| Opioids+antidepressants | 39.8 | 4.5 | 7 |
| Opioids+antiepileptics | 57.9 | 10.3 | 5.2 |
| Opioids+neurotropin | 10.2 | 2.9 | 10 |
| Spicamycin Derivative | 55.4 | 3.6 | 5.5 |

**Figure S1A network meta-analysis results of pain relief**

Treatments are ranked according to their surface under the curve cumulative ranking (SUCRA) for pain relief starting with the best. Comparisons between treatments should be read from left to right and the estimate is in the cell in common between the column-defining treatment and the row-defining treatment. Dichotomous outcomes are reported as risk ratios (RRs). In the left lower half, risk ratios (RRs) upeer than 1 favor the column-defining treatment, Cells in bold print indicate significant results. Drug abbreviations: ON= Opioids+neurotropin, SD= Spicamycin Derivative,VB12=Vitamin B12.

**Figure S1B network meta-analysis results of nausea and vomiting**

Treatments are ranked according to their surface under the curve cumulative ranking (SUCRA) for nausea and vomiting starting with the best. Comparisons between treatments should be read from left to right and the estimate is in the cell in common between the column-defining treatment and the row-defining treatment. Dichotomous outcomes are reported as risk ratios (RRs). In the left lower half, risk ratios (RRs) lower than 1 favor the column-defining treatment, Cells in bold print indicate significant results. Drug abbreviations: VB12=Vitamin B12, AA= Antiepileptics+antidepressants, OAntidep= Opioids+antidepressants , ON= Opioids+neurotropin, OAntiepi= Opioids+antiepileptics, OAN= Opioids+antiepileptics+NSAIDs, OAA= Opioids+antiepileptics+antidepressants.

**Figure S1C network meta-analysis results of overall adverse events**

Treatments are ranked according to their surface under the curve cumulative ranking (SUCRA) for overall adverse events starting with the best. Comparisons between treatments should be read from left to right and the estimate is in the cell in common between the column-defining treatment and the row-defining treatment. Dichotomous outcomes are reported as risk ratios (RRs). In the left lower half, risk ratios (RRs) lower than 1 favor the column-defining treatment, Cells in bold print indicate significant results. Drug abbreviations: KA= Anaesthetics+antidepressants, OAntiepi= Opioids+antiepileptics, SD= Spicamycin Derivative, OAntidep= Opioids+antidepressants, ON= Opioids+neurotropin.

**Table S5 global inconsistency**

| Testing for inconsistency: | pain intensity | pain relief | nausea and vomiting | overall adverse events |
| --- | --- | --- | --- | --- |
| chi2 | chi2( 5) = 3.45 | - | chi2( 2) = 1.01 | chi2( 1) = 1.54 |
| Prob > chi2 | Prob > chi2 = 0.6317 | - | Prob > chi2 = 0.6026 | Prob > chi2 = 0.2141 |

**Figure S2 Node inconsistency of All Outcomes**

**Figure S2A Node inconsistency of pain intensity**

**Figure S2B Node inconsistency of pain relief**

**Figure S2C Node inconsistency of nausea and vomiting**

**Figure S2D Node inconsistency of overall adverse events**

**Figure S3 Funnel Plots of All Outcomes**

**Figure S3A，Funnel Plots of pain intensity**

**Legend**: In the comparison-adjusted funnel plot, the horizontal axis shows the difference of each i-study estimate YiXY from the summary effect for the respective comparison (YiXY-μXY) while the vertical axis presents the measure of dispersion of YiXY, namely the standard error of the effect size. The red line shows the null hypothesis. Each point represents a direct comparison; different colors correspond to different comparisons. The dashed black line represents the 95% confidence interval. The purple line represents the regression line; the regression line demonstrates that no asymmetry is present. A, Placebo; B, Anaesthetics; C, Antidepressants; D, Antiepileptics; E, Antiepileptics+Antidepressants; F, Costus; G, Crocin; H, Anaesthetics+antidepressants; I, LC07; J, Nabiximols; K, Opioids; L, Opioids+NSAIDs; M,Opioids+Antidepressants; N, Opioids+Antiepileptics; O, Opioids+Antiepileptics+Antidepressants; P, Opioids+Antiepileptics+NSAIDs; Q, Opioids+Neurotropin; R, Spicamycin Derivative; S, Vitamin B12.

**Figure S3B，Funnel Plots of pain relief**

Legend: In the comparison-adjusted funnel plot, the horizontal axis shows the difference of each i-study estimate YiXY from the summary effect for the respective comparison (YiXY-μXY) while the vertical axis presents the measure of dispersion of YiXY, namely the standard error of the effect size. The red line shows the null hypothesis. Each point represents a direct comparison; different colors correspond to different comparisons. The dashed black line represents the 95% confidence interval. The brown line represents the regression line; the regression line demonstrates that no asymmetry is present. A,Placebo; B,Anaesthetics; C,Antidepressants; D,Antiepileptics; E,Opioids; F,Opioids+Neurotropin; G,Spicamycin Derivative; H,Vitamin B12.

**Figure S3C，Funnel Plots of nausea and vomiting**

Legend: In the comparison-adjusted funnel plot, the horizontal axis shows the difference of each i-study estimate YiXY from the summary effect for the respective comparison (YiXY-μXY) while the vertical axis presents the measure of dispersion of YiXY, namely the standard error of the effect size. The red line shows the null hypothesis. Each point represents a direct comparison; different colors correspond to different comparisons. The dashed black line represents the 95% confidence interval. The blue line represents the regression line; the regression line demonstrates that no asymmetry is present..A,Placebo; B,Antidepressants; C,Antiepileptics; D,Antiepileptics+Antidepressants; E,Crocin; F,Nabiximols; G,Opioids; H,Opioids+Antidepressants; I,Opioids+Antiepileptics; J,Opioids+antiepileptics+antidepressants; K,Opioids+Antiepileptics+NSAIDs; L,Opioids+Neurotropin; M,Vitamin B12.

**Figure S3D，Funnel Plots of overall adverse events**

Legend: In the comparison-adjusted funnel plot, the horizontal axis shows the difference of each i-study estimate YiXY from the summary effect for the respective comparison (YiXY-μXY) while the vertical axis presents the measure of dispersion of YiXY, namely the standard error of the effect size. The red line shows the null hypothesis. Each point represents a direct comparison; different colors correspond to different comparisons. The dashed black line represents the 95% confidence interval. The dark pink line represents the regression line; the regression line demonstrates that no asymmetry is present. A,Placebo; B,Anaesthetics; C,Antidepressants; D,Antiepileptics; E,Costus; F,Anesthetics+antidepressants; G,Opioids; H,Opioids+Antidepressants; I,Opioids+Antiepileptics; J,Opioids+Neurotropin; K,Spicamycin Derivative.

**Table S6 Grading the evidence of the network meta-analysis using CINeMA.**

**Table S6A CINeMA for the primary outcome pian intensity**

| Comparison | Number of studies | Within-study bias | Reporting bias | Indirectness | Imprecision | Heterogeneity | Incoherence | Confidence rating | Reason(s) for downgrading |
| --- | --- | --- | --- | --- | --- | --- | --- | --- | --- |
| Anaesthetics:Placebo | 4 | No concerns | Low risk | No concerns | No concerns | Some concerns | No concerns | Moderate | "Heterogeneity" |
| Anaesthetics+antidepressants:Placebo | 1 | No concerns | Low risk | No concerns | Major concerns | No concerns | No concerns | Low | "Imprecision" |
| Antidepressants:Antiepileptics | 4 | No concerns | Low risk | No concerns | Some concerns | Some concerns | No concerns | Low | "Imprecision","Heterogeneity" |
| Antidepressants:Antiepileptics+Antidepressants | 1 | No concerns | Low risk | No concerns | No concerns | No concerns | No concerns | High |  |
| Antidepressants:Placebo | 5 | No concerns | Low risk | No concerns | Some concerns | Some concerns | No concerns | Low | "Imprecision","Heterogeneity" |
| Antidepressants:Vitamin B12 | 1 | Some concerns | Low risk | No concerns | No concerns | Some concerns | No concerns | Low | "Within-study bias","Heterogeneity" |
| Antiepileptics:Antiepileptics+Antidepressants | 1 | No concerns | Low risk | No concerns | No concerns | No concerns | No concerns | High |  |
| Antiepileptics:Opioids | 1 | No concerns | Low risk | No concerns | Some concerns | Some concerns | Some concerns | Very low | "Imprecision","Heterogeneity","Incoherence" |
| Antiepileptics:Placebo | 5 | No concerns | Low risk | No concerns | No concerns | Major concerns | No concerns | Low | "Heterogeneity" |
| Costus:Placebo | 1 | No concerns | Low risk | No concerns | Some concerns | No concerns | No concerns | Moderate | "Imprecision" |
| Crocin:Placebo | 1 | No concerns | Low risk | No concerns | No concerns | Some concerns | No concerns | Moderate | "Heterogeneity" |
| LC07:Placebo | 1 | Some concerns | Low risk | No concerns | No concerns | Some concerns | No concerns | Low | "Within-study bias","Heterogeneity" |
| Nabiximols:Placebo | 1 | No concerns | Low risk | No concerns | Major concerns | No concerns | No concerns | Low | "Imprecision" |
| Opioids:Opioids+Antiepileptics | 2 | Major concerns | Low risk | No concerns | No concerns | Some concerns | No concerns | Very low | "Within-study bias","Heterogeneity" |
| Opioids:Opioids+NSAIDs | 1 | No concerns | Low risk | No concerns | Major concerns | No concerns | No concerns | Low | "Imprecision" |
| Opioids:Opioids+Neurotropin | 2 | Some concerns | Low risk | No concerns | No concerns | Some concerns | No concerns | Low | "Within-study bias","Heterogeneity" |
| Opioids:Placebo | 1 | No concerns | Low risk | No concerns | Major concerns | No concerns | Major concerns | Very low | "Imprecision","Incoherence" |
| Opioids+Antidepressants:Opioids+Antiepileptics | 1 | No concerns | Low risk | No concerns | Major concerns | No concerns | No concerns | Low | "Imprecision" |
| Opioids+Antiepileptics:Opioids+Antiepileptics+Antidepressants | 1 | No concerns | Low risk | No concerns | Major concerns | No concerns | No concerns | Low | "Imprecision" |
| Opioids+Antiepileptics:Opioids+Antiepileptics+NSAIDs | 1 | Some concerns | Low risk | No concerns | No concerns | Some concerns | No concerns | Low | "Within-study bias","Heterogeneity" |
| Placebo:Spicamycin Derivative | 1 | No concerns | Low risk | No concerns | No concerns | Some concerns | No concerns | Moderate | "Heterogeneity" |
| Anaesthetics:Anaesthetics+antidepressants | 0 | No concerns | Low risk | No concerns | Some concerns | Some concerns | No concerns | Low | "Imprecision","Heterogeneity" |
| Anaesthetics:Antidepressants | 0 | No concerns | Low risk | No concerns | Some concerns | Some concerns | No concerns | Low | "Imprecision","Heterogeneity" |
| Anaesthetics:Antiepileptics | 0 | No concerns | Low risk | No concerns | Some concerns | Some concerns | No concerns | Low | "Imprecision","Heterogeneity" |
| Anaesthetics:Antiepileptics+Antidepressants | 0 | No concerns | Low risk | No concerns | Some concerns | No concerns | No concerns | Moderate | "Imprecision" |
| Anaesthetics:Costus | 0 | No concerns | Low risk | No concerns | Major concerns | No concerns | No concerns | Low | "Imprecision" |
| Anaesthetics:Crocin | 0 | No concerns | Low risk | No concerns | Major concerns | No concerns | No concerns | Low | "Imprecision" |
| Anaesthetics:LC07 | 0 | Some concerns | Low risk | No concerns | Major concerns | No concerns | No concerns | Very low | "Within-study bias","Imprecision" |
| Anaesthetics:Nabiximols | 0 | No concerns | Low risk | No concerns | Major concerns | No concerns | No concerns | Low | "Imprecision" |
| Anaesthetics:Opioids | 0 | No concerns | Low risk | No concerns | Some concerns | No concerns | No concerns | Moderate | "Imprecision" |
| Anaesthetics:Opioids+Antidepressants | 0 | No concerns | Low risk | No concerns | Major concerns | No concerns | No concerns | Low | "Imprecision" |
| Anaesthetics:Opioids+Antiepileptics | 0 | Some concerns | Low risk | No concerns | Major concerns | No concerns | No concerns | Very low | "Within-study bias","Imprecision" |
| Anaesthetics:Opioids+Antiepileptics+Antidepressants | 0 | No concerns | Low risk | No concerns | Major concerns | No concerns | No concerns | Low | "Imprecision" |
| Anaesthetics:Opioids+Antiepileptics+NSAIDs | 0 | Some concerns | Low risk | No concerns | Some concerns | Some concerns | No concerns | Very low | "Within-study bias","Imprecision","Heterogeneity" |
| Anaesthetics:Opioids+NSAIDs | 0 | No concerns | Low risk | No concerns | Some concerns | Some concerns | No concerns | Low | "Imprecision","Heterogeneity" |
| Anaesthetics:Opioids+Neurotropin | 0 | No concerns | Low risk | No concerns | Major concerns | No concerns | No concerns | Low | "Imprecision" |
| Anaesthetics:Spicamycin Derivative | 0 | No concerns | Low risk | No concerns | Major concerns | No concerns | No concerns | Low | "Imprecision" |
| Anaesthetics:Vitamin B12 | 0 | Some concerns | Low risk | No concerns | No concerns | No concerns | No concerns | Moderate | "Within-study bias" |
| Anaesthetics+antidepressants:Antidepressants | 0 | No concerns | Low risk | No concerns | Major concerns | No concerns | No concerns | Low | "Imprecision" |
| Anaesthetics+antidepressants:Antiepileptics | 0 | No concerns | Low risk | No concerns | Major concerns | No concerns | No concerns | Low | "Imprecision" |
| Anaesthetics+antidepressants:Antiepileptics+Antidepressants | 0 | No concerns | Low risk | No concerns | No concerns | Some concerns | No concerns | Moderate | "Heterogeneity" |
| Anaesthetics+antidepressants:Costus | 0 | No concerns | Low risk | No concerns | Major concerns | No concerns | No concerns | Low | "Imprecision" |
| Anaesthetics+antidepressants:Crocin | 0 | No concerns | Low risk | No concerns | Some concerns | Some concerns | No concerns | Low | "Imprecision","Heterogeneity" |
| Anaesthetics+antidepressants:LC07 | 0 | Some concerns | Low risk | No concerns | Some concerns | Some concerns | No concerns | Very low | "Within-study bias","Imprecision","Heterogeneity" |
| Anaesthetics+antidepressants:Nabiximols | 0 | No concerns | Low risk | No concerns | Major concerns | No concerns | No concerns | Low | "Imprecision" |
| Anaesthetics+antidepressants:Opioids | 0 | No concerns | Low risk | No concerns | Major concerns | No concerns | No concerns | Low | "Imprecision" |
| Anaesthetics+antidepressants:Opioids+Antidepressants | 0 | No concerns | Low risk | No concerns | Major concerns | No concerns | No concerns | Low | "Imprecision" |
| Anaesthetics+antidepressants:Opioids+Antiepileptics | 0 | No concerns | Low risk | No concerns | Major concerns | No concerns | No concerns | Low | "Imprecision" |
| Anaesthetics+antidepressants:Opioids+Antiepileptics+Antidepressants | 0 | No concerns | Low risk | No concerns | Major concerns | No concerns | No concerns | Low | "Imprecision" |
| Anaesthetics+antidepressants:Opioids+Antiepileptics+NSAIDs | 0 | Some concerns | Low risk | No concerns | No concerns | Some concerns | No concerns | Low | "Within-study bias","Heterogeneity" |
| Anaesthetics+antidepressants:Opioids+NSAIDs | 0 | No concerns | Low risk | No concerns | Major concerns | No concerns | No concerns | Low | "Imprecision" |
| Anaesthetics+antidepressants:Opioids+Neurotropin | 0 | No concerns | Low risk | No concerns | Major concerns | No concerns | No concerns | Low | "Imprecision" |
| Anaesthetics+antidepressants:Spicamycin Derivative | 0 | No concerns | Low risk | No concerns | Some concerns | Some concerns | No concerns | Low | "Imprecision","Heterogeneity" |
| Anaesthetics+antidepressants:Vitamin B12 | 0 | No concerns | Low risk | No concerns | Some concerns | Some concerns | No concerns | Low | "Imprecision","Heterogeneity" |
| Antidepressants:Costus | 0 | No concerns | Low risk | No concerns | Some concerns | Some concerns | No concerns | Low | "Imprecision","Heterogeneity" |
| Antidepressants:Crocin | 0 | No concerns | Low risk | No concerns | Some concerns | Some concerns | No concerns | Low | "Imprecision","Heterogeneity" |
| Antidepressants:LC07 | 0 | Some concerns | Low risk | No concerns | Some concerns | No concerns | No concerns | Low | "Within-study bias","Imprecision" |
| Antidepressants:Nabiximols | 0 | No concerns | Low risk | No concerns | Major concerns | No concerns | No concerns | Low | "Imprecision" |
| Antidepressants:Opioids | 0 | No concerns | Low risk | No concerns | Some concerns | Some concerns | No concerns | Low | "Imprecision","Heterogeneity" |
| Antidepressants:Opioids+Antidepressants | 0 | No concerns | Low risk | No concerns | Major concerns | No concerns | No concerns | Low | "Imprecision" |
| Antidepressants:Opioids+Antiepileptics | 0 | Some concerns | Low risk | No concerns | Some concerns | Some concerns | No concerns | Very low | "Within-study bias","Imprecision","Heterogeneity" |
| Antidepressants:Opioids+Antiepileptics+Antidepressants | 0 | No concerns | Low risk | No concerns | Major concerns | No concerns | No concerns | Low | "Imprecision" |
| Antidepressants:Opioids+Antiepileptics+NSAIDs | 0 | Some concerns | Low risk | No concerns | No concerns | Some concerns | No concerns | Low | "Within-study bias","Heterogeneity" |
| Antidepressants:Opioids+NSAIDs | 0 | No concerns | Low risk | No concerns | Major concerns | No concerns | No concerns | Low | "Imprecision" |
| Antidepressants:Opioids+Neurotropin | 0 | No concerns | Low risk | No concerns | Some concerns | Some concerns | No concerns | Low | "Imprecision","Heterogeneity" |
| Antidepressants:Spicamycin Derivative | 0 | No concerns | Low risk | No concerns | Some concerns | Some concerns | No concerns | Low | "Imprecision","Heterogeneity" |
| Antiepileptics:Costus | 0 | No concerns | Low risk | No concerns | Major concerns | No concerns | No concerns | Low | "Imprecision" |
| Antiepileptics:Crocin | 0 | No concerns | Low risk | No concerns | Some concerns | Some concerns | No concerns | Low | "Imprecision","Heterogeneity" |
| Antiepileptics:LC07 | 0 | Some concerns | Low risk | No concerns | Some concerns | Some concerns | No concerns | Very low | "Within-study bias","Imprecision","Heterogeneity" |
| Antiepileptics:Nabiximols | 0 | No concerns | Low risk | No concerns | Major concerns | No concerns | No concerns | Low | "Imprecision" |
| Antiepileptics:Opioids+Antidepressants | 0 | No concerns | Low risk | No concerns | Major concerns | No concerns | No concerns | Low | "Imprecision" |
| Antiepileptics:Opioids+Antiepileptics | 0 | Some concerns | Low risk | No concerns | Major concerns | No concerns | No concerns | Very low | "Within-study bias","Imprecision" |
| Antiepileptics:Opioids+Antiepileptics+Antidepressants | 0 | No concerns | Low risk | No concerns | Major concerns | No concerns | No concerns | Low | "Imprecision" |
| Antiepileptics:Opioids+Antiepileptics+NSAIDs | 0 | Some concerns | Low risk | No concerns | No concerns | Some concerns | No concerns | Low | "Within-study bias","Heterogeneity" |
| Antiepileptics:Opioids+NSAIDs | 0 | No concerns | Low risk | No concerns | Major concerns | No concerns | No concerns | Low | "Imprecision" |
| Antiepileptics:Opioids+Neurotropin | 0 | No concerns | Low risk | No concerns | Major concerns | No concerns | No concerns | Low | "Imprecision" |
| Antiepileptics:Spicamycin Derivative | 0 | No concerns | Low risk | No concerns | Some concerns | Some concerns | No concerns | Low | "Imprecision","Heterogeneity" |
| Antiepileptics:Vitamin B12 | 0 | Some concerns | Low risk | No concerns | No concerns | Some concerns | No concerns | Low | "Within-study bias","Heterogeneity" |
| Antiepileptics+Antidepressants:Costus | 0 | No concerns | Low risk | No concerns | Major concerns | No concerns | No concerns | Low | "Imprecision" |
| Antiepileptics+Antidepressants:Crocin | 0 | No concerns | Low risk | No concerns | Major concerns | No concerns | No concerns | Low | "Imprecision" |
| Antiepileptics+Antidepressants:LC07 | 0 | No concerns | Low risk | No concerns | Major concerns | No concerns | No concerns | Low | "Imprecision" |
| Antiepileptics+Antidepressants:Nabiximols | 0 | No concerns | Low risk | No concerns | No concerns | Some concerns | No concerns | Moderate | "Heterogeneity" |
| Antiepileptics+Antidepressants:Opioids | 0 | No concerns | Low risk | No concerns | No concerns | No concerns | No concerns | High |  |
| Antiepileptics+Antidepressants:Opioids+Antidepressants | 0 | No concerns | Low risk | No concerns | Some concerns | Some concerns | No concerns | Low | "Imprecision","Heterogeneity" |
| Antiepileptics+Antidepressants:Opioids+Antiepileptics | 0 | No concerns | Low risk | No concerns | Some concerns | Some concerns | No concerns | Low | "Imprecision","Heterogeneity" |
| Antiepileptics+Antidepressants:Opioids+Antiepileptics+Antidepressants | 0 | No concerns | Low risk | No concerns | Major concerns | No concerns | No concerns | Low | "Imprecision" |
| Antiepileptics+Antidepressants:Opioids+Antiepileptics+NSAIDs | 0 | Some concerns | Low risk | No concerns | Major concerns | No concerns | No concerns | Very low | "Within-study bias","Imprecision" |
| Antiepileptics+Antidepressants:Opioids+NSAIDs | 0 | No concerns | Low risk | No concerns | No concerns | No concerns | No concerns | High |  |
| Antiepileptics+Antidepressants:Opioids+Neurotropin | 0 | No concerns | Low risk | No concerns | Some concerns | Some concerns | No concerns | Low | "Imprecision","Heterogeneity" |
| Antiepileptics+Antidepressants:Placebo | 0 | No concerns | Low risk | No concerns | No concerns | No concerns | No concerns | High |  |
| Antiepileptics+Antidepressants:Spicamycin Derivative | 0 | No concerns | Low risk | No concerns | Major concerns | No concerns | No concerns | Low | "Imprecision" |
| Antiepileptics+Antidepressants:Vitamin B12 | 0 | No concerns | Low risk | No concerns | No concerns | No concerns | No concerns | High |  |
| Costus:Crocin | 0 | No concerns | Low risk | No concerns | Major concerns | No concerns | No concerns | Low | "Imprecision" |
| Costus:LC07 | 0 | Some concerns | Low risk | No concerns | Major concerns | No concerns | No concerns | Very low | "Within-study bias","Imprecision" |
| Costus:Nabiximols | 0 | No concerns | Low risk | No concerns | Major concerns | No concerns | No concerns | Low | "Imprecision" |
| Costus:Opioids | 0 | No concerns | Low risk | No concerns | Some concerns | Some concerns | No concerns | Low | "Imprecision","Heterogeneity" |
| Costus:Opioids+Antidepressants | 0 | No concerns | Low risk | No concerns | Major concerns | No concerns | No concerns | Low | "Imprecision" |
| Costus:Opioids+Antiepileptics | 0 | No concerns | Low risk | No concerns | Major concerns | No concerns | No concerns | Low | "Imprecision" |
| Costus:Opioids+Antiepileptics+Antidepressants | 0 | No concerns | Low risk | No concerns | Major concerns | No concerns | No concerns | Low | "Imprecision" |
| Costus:Opioids+Antiepileptics+NSAIDs | 0 | Some concerns | Low risk | No concerns | Major concerns | No concerns | No concerns | Very low | "Within-study bias","Imprecision" |
| Costus:Opioids+NSAIDs | 0 | No concerns | Low risk | No concerns | Some concerns | Some concerns | No concerns | Low | "Imprecision","Heterogeneity" |
| Costus:Opioids+Neurotropin | 0 | No concerns | Low risk | No concerns | Major concerns | No concerns | No concerns | Low | "Imprecision" |
| Costus:Spicamycin Derivative | 0 | No concerns | Low risk | No concerns | Major concerns | No concerns | No concerns | Low | "Imprecision" |
| Costus:Vitamin B12 | 0 | No concerns | Low risk | No concerns | No concerns | No concerns | No concerns | High |  |
| Crocin:LC07 | 0 | Some concerns | Low risk | No concerns | Major concerns | No concerns | No concerns | Very low | "Within-study bias","Imprecision" |
| Crocin:Nabiximols | 0 | No concerns | Low risk | No concerns | Major concerns | No concerns | No concerns | Low | "Imprecision" |
| Crocin:Opioids | 0 | No concerns | Low risk | No concerns | Some concerns | No concerns | No concerns | Moderate | "Imprecision" |
| Crocin:Opioids+Antidepressants | 0 | No concerns | Low risk | No concerns | Major concerns | No concerns | No concerns | Low | "Imprecision" |
| Crocin:Opioids+Antiepileptics | 0 | No concerns | Low risk | No concerns | Major concerns | No concerns | No concerns | Low | "Imprecision" |
| Crocin:Opioids+Antiepileptics+Antidepressants | 0 | No concerns | Low risk | No concerns | Major concerns | No concerns | No concerns | Low | "Imprecision" |
| Crocin:Opioids+Antiepileptics+NSAIDs | 0 | Some concerns | Low risk | No concerns | Major concerns | No concerns | No concerns | Very low | "Within-study bias","Imprecision" |
| Crocin:Opioids+NSAIDs | 0 | No concerns | Low risk | No concerns | Some concerns | Some concerns | No concerns | Low | "Imprecision","Heterogeneity" |
| Crocin:Opioids+Neurotropin | 0 | No concerns | Low risk | No concerns | Major concerns | No concerns | No concerns | Low | "Imprecision" |
| Crocin:Spicamycin Derivative | 0 | No concerns | Low risk | No concerns | Major concerns | No concerns | No concerns | Low | "Imprecision" |
| Crocin:Vitamin B12 | 0 | No concerns | Low risk | No concerns | No concerns | No concerns | No concerns | High |  |
| LC07:Nabiximols | 0 | Some concerns | Low risk | No concerns | Some concerns | Some concerns | No concerns | Very low | "Within-study bias","Imprecision","Heterogeneity" |
| LC07:Opioids | 0 | No concerns | Low risk | No concerns | No concerns | Some concerns | No concerns | Moderate | "Heterogeneity" |
| LC07:Opioids+Antidepressants | 0 | Some concerns | Low risk | No concerns | Major concerns | No concerns | No concerns | Very low | "Within-study bias","Imprecision" |
| LC07:Opioids+Antiepileptics | 0 | Some concerns | Low risk | No concerns | Major concerns | No concerns | No concerns | Very low | "Within-study bias","Imprecision" |
| LC07:Opioids+Antiepileptics+Antidepressants | 0 | Some concerns | Low risk | No concerns | Major concerns | No concerns | No concerns | Very low | "Within-study bias","Imprecision" |
| LC07:Opioids+Antiepileptics+NSAIDs | 0 | Some concerns | Low risk | No concerns | Major concerns | No concerns | No concerns | Very low | "Within-study bias","Imprecision" |
| LC07:Opioids+NSAIDs | 0 | No concerns | Low risk | No concerns | Some concerns | Some concerns | No concerns | Low | "Imprecision","Heterogeneity" |
| LC07:Opioids+Neurotropin | 0 | Some concerns | Low risk | No concerns | Major concerns | No concerns | No concerns | Very low | "Within-study bias","Imprecision" |
| LC07:Spicamycin Derivative | 0 | Some concerns | Low risk | No concerns | Major concerns | No concerns | No concerns | Very low | "Within-study bias","Imprecision" |
| LC07:Vitamin B12 | 0 | Some concerns | Low risk | No concerns | No concerns | No concerns | No concerns | Moderate | "Within-study bias" |
| Nabiximols:Opioids | 0 | No concerns | Low risk | No concerns | Major concerns | No concerns | No concerns | Low | "Imprecision" |
| Nabiximols:Opioids+Antidepressants | 0 | No concerns | Low risk | No concerns | Major concerns | No concerns | No concerns | Low | "Imprecision" |
| Nabiximols:Opioids+Antiepileptics | 0 | No concerns | Low risk | No concerns | Major concerns | No concerns | No concerns | Low | "Imprecision" |
| Nabiximols:Opioids+Antiepileptics+Antidepressants | 0 | No concerns | Low risk | No concerns | Major concerns | No concerns | No concerns | Low | "Imprecision" |
| Nabiximols:Opioids+Antiepileptics+NSAIDs | 0 | Some concerns | Low risk | No concerns | Some concerns | No concerns | No concerns | Low | "Within-study bias","Imprecision" |
| Nabiximols:Opioids+NSAIDs | 0 | No concerns | Low risk | No concerns | Major concerns | No concerns | No concerns | Low | "Imprecision" |
| Nabiximols:Opioids+Neurotropin | 0 | No concerns | Low risk | No concerns | Major concerns | No concerns | No concerns | Low | "Imprecision" |
| Nabiximols:Spicamycin Derivative | 0 | No concerns | Low risk | No concerns | Major concerns | No concerns | No concerns | Low | "Imprecision" |
| Nabiximols:Vitamin B12 | 0 | No concerns | Low risk | No concerns | Some concerns | Some concerns | No concerns | Low | "Imprecision","Heterogeneity" |
| Opioids:Opioids+Antidepressants | 0 | Some concerns | Low risk | No concerns | Some concerns | Some concerns | No concerns | Very low | "Within-study bias","Imprecision","Heterogeneity" |
| Opioids:Opioids+Antiepileptics+Antidepressants | 0 | Some concerns | Low risk | No concerns | Some concerns | No concerns | No concerns | Low | "Within-study bias","Imprecision" |
| Opioids:Opioids+Antiepileptics+NSAIDs | 0 | Some concerns | Low risk | No concerns | No concerns | No concerns | No concerns | Moderate | "Within-study bias" |
| Opioids:Spicamycin Derivative | 0 | No concerns | Low risk | No concerns | Some concerns | No concerns | No concerns | Moderate | "Imprecision" |
| Opioids:Vitamin B12 | 0 | No concerns | Low risk | No concerns | Some concerns | Some concerns | No concerns | Low | "Imprecision","Heterogeneity" |
| Opioids+Antidepressants:Opioids+Antiepileptics+Antidepressants | 0 | No concerns | Low risk | No concerns | Major concerns | No concerns | No concerns | Low | "Imprecision" |
| Opioids+Antidepressants:Opioids+Antiepileptics+NSAIDs | 0 | Some concerns | Low risk | No concerns | Some concerns | No concerns | No concerns | Low | "Within-study bias","Imprecision" |
| Opioids+Antidepressants:Opioids+NSAIDs | 0 | Some concerns | Low risk | No concerns | Major concerns | No concerns | No concerns | Very low | "Within-study bias","Imprecision" |
| Opioids+Antidepressants:Opioids+Neurotropin | 0 | Some concerns | Low risk | No concerns | Major concerns | No concerns | No concerns | Very low | "Within-study bias","Imprecision" |
| Opioids+Antidepressants:Placebo | 0 | No concerns | Low risk | No concerns | Major concerns | No concerns | No concerns | Low | "Imprecision" |
| Opioids+Antidepressants:Spicamycin Derivative | 0 | No concerns | Low risk | No concerns | Major concerns | No concerns | No concerns | Low | "Imprecision" |
| Opioids+Antidepressants:Vitamin B12 | 0 | Some concerns | Low risk | No concerns | No concerns | Some concerns | No concerns | Low | "Within-study bias","Heterogeneity" |
| Opioids+Antiepileptics:Opioids+NSAIDs | 0 | Some concerns | Low risk | No concerns | Some concerns | Some concerns | No concerns | Very low | "Within-study bias","Imprecision","Heterogeneity" |
| Opioids+Antiepileptics:Opioids+Neurotropin | 0 | Some concerns | Low risk | No concerns | Major concerns | No concerns | No concerns | Very low | "Within-study bias","Imprecision" |
| Opioids+Antiepileptics:Placebo | 0 | Some concerns | Low risk | No concerns | Some concerns | Some concerns | No concerns | Very low | "Within-study bias","Imprecision","Heterogeneity" |
| Opioids+Antiepileptics:Spicamycin Derivative | 0 | No concerns | Low risk | No concerns | Major concerns | No concerns | No concerns | Low | "Imprecision" |
| Opioids+Antiepileptics:Vitamin B12 | 0 | Some concerns | Low risk | No concerns | No concerns | Some concerns | No concerns | Low | "Within-study bias","Heterogeneity" |
| Opioids+Antiepileptics+Antidepressants:Opioids+Antiepileptics+NSAIDs | 0 | Some concerns | Low risk | No concerns | Major concerns | No concerns | No concerns | Very low | "Within-study bias","Imprecision" |
| Opioids+Antiepileptics+Antidepressants:Opioids+NSAIDs | 0 | Some concerns | Low risk | No concerns | Some concerns | Some concerns | No concerns | Very low | "Within-study bias","Imprecision","Heterogeneity" |

**Table S6B CINeMA for pain relief**

| Comparison | Number of studies | Within-study bias | Reporting bias | Indirectness | Imprecision | Heterogeneity | Incoherence | Confidence rating | Reason(s) for downgrading |
| --- | --- | --- | --- | --- | --- | --- | --- | --- | --- |
| Anaesthetics:Placebo | 2 | No concerns | Low risk | No concerns | Major concerns | No concerns | Major concerns | Low | "Imprecision","Incoherence" |
| Antidepressants:Placebo | 1 | No concerns | Low risk | No concerns | Major concerns | No concerns | Major concerns | Low | "Imprecision","Incoherence" |
| Antidepressants:Vitamin B12 | 1 | Some concerns | Low risk | No concerns | Some concerns | Some concerns | Major concerns | Very low | "Within-study bias","Imprecision","Heterogeneity","Incoherence" |
| Antiepileptics:Opioids | 1 | No concerns | Low risk | No concerns | Major concerns | No concerns | Major concerns | Low | "Imprecision","Incoherence" |
| Antiepileptics:Placebo | 1 | No concerns | Low risk | No concerns | Major concerns | No concerns | Major concerns | Low | "Imprecision","Incoherence" |
| Opioids:Opioids+Neurotropin | 1 | Some concerns | Low risk | No concerns | Some concerns | Some concerns | Major concerns | Very low | "Within-study bias","Imprecision","Heterogeneity","Incoherence" |
| Placebo:Spicamycin Derivative | 1 | No concerns | Low risk | No concerns | Major concerns | No concerns | Major concerns | Low | "Imprecision","Incoherence" |
| Anaesthetics:Antidepressants | 0 | No concerns | Low risk | No concerns | Major concerns | No concerns | Major concerns | Low | "Imprecision","Incoherence" |
| Anaesthetics:Antiepileptics | 0 | No concerns | Low risk | No concerns | Major concerns | No concerns | Major concerns | Low | "Imprecision","Incoherence" |
| Anaesthetics:Opioids | 0 | No concerns | Low risk | No concerns | Major concerns | No concerns | Major concerns | Low | "Imprecision","Incoherence" |
| Anaesthetics:Opioids+Neurotropin | 0 | No concerns | Low risk | No concerns | Major concerns | No concerns | Major concerns | Low | "Imprecision","Incoherence" |
| Anaesthetics:Spicamycin Derivative | 0 | No concerns | Low risk | No concerns | Major concerns | No concerns | Major concerns | Low | "Imprecision","Incoherence" |
| Anaesthetics:Vitamin B12 | 0 | No concerns | Low risk | No concerns | Major concerns | No concerns | Major concerns | Low | "Imprecision","Incoherence" |
| Antidepressants:Antiepileptics | 0 | No concerns | Low risk | No concerns | Major concerns | No concerns | Major concerns | Low | "Imprecision","Incoherence" |
| Antidepressants:Opioids | 0 | No concerns | Low risk | No concerns | Major concerns | No concerns | Major concerns | Low | "Imprecision","Incoherence" |
| Antidepressants:Opioids+Neurotropin | 0 | No concerns | Low risk | No concerns | Major concerns | No concerns | Major concerns | Low | "Imprecision","Incoherence" |
| Antidepressants:Spicamycin Derivative | 0 | No concerns | Low risk | No concerns | Major concerns | No concerns | Major concerns | Low | "Imprecision","Incoherence" |
| Antiepileptics:Opioids+Neurotropin | 0 | Some concerns | Low risk | No concerns | Major concerns | No concerns | Major concerns | Very low | "Within-study bias","Imprecision","Incoherence" |
| Antiepileptics:Spicamycin Derivative | 0 | No concerns | Low risk | No concerns | Major concerns | No concerns | Major concerns | Low | "Imprecision","Incoherence" |
| Antiepileptics:Vitamin B12 | 0 | No concerns | Low risk | No concerns | Major concerns | No concerns | Major concerns | Low | "Imprecision","Incoherence" |
| Opioids:Placebo | 0 | No concerns | Low risk | No concerns | Major concerns | No concerns | Major concerns | Low | "Imprecision","Incoherence" |
| Opioids:Spicamycin Derivative | 0 | No concerns | Low risk | No concerns | Major concerns | No concerns | Major concerns | Low | "Imprecision","Incoherence" |
| Opioids:Vitamin B12 | 0 | No concerns | Low risk | No concerns | Major concerns | No concerns | Major concerns | Low | "Imprecision","Incoherence" |
| Opioids+Neurotropin:Placebo | 0 | No concerns | Low risk | No concerns | Major concerns | No concerns | Major concerns | Low | "Imprecision","Incoherence" |
| Opioids+Neurotropin:Spicamycin Derivative | 0 | No concerns | Low risk | No concerns | Major concerns | No concerns | Major concerns | Low | "Imprecision","Incoherence" |
| Opioids+Neurotropin:Vitamin B12 | 0 | No concerns | Low risk | No concerns | Major concerns | No concerns | Major concerns | Low | "Imprecision","Incoherence" |
| Placebo:Vitamin B12 | 0 | Some concerns | Low risk | No concerns | Major concerns | No concerns | Major concerns | Very low | "Within-study bias","Imprecision","Incoherence" |
| Spicamycin Derivative:Vitamin B12 | 0 | No concerns | Low risk | No concerns | Major concerns | No concerns | Major concerns | Low | "Imprecision","Incoherence" |

**Table S6C CINeMA for nausea and vomiting**

| Comparison | Number of studies | Within-study bias | Reporting bias | Indirectness | Imprecision | Heterogeneity | Incoherence | Confidence rating | Reason(s) for downgrading |
| --- | --- | --- | --- | --- | --- | --- | --- | --- | --- |
| Antidepressants:Antiepileptics | 2 | No concerns | Low risk | No concerns | Major concerns | No concerns | No concerns | Low | "Imprecision" |
| Antidepressants:Antiepileptics+Antidepressants | 1 | No concerns | Low risk | No concerns | Major concerns | No concerns | No concerns | Low | "Imprecision" |
| Antidepressants:Placebo | 3 | No concerns | Low risk | No concerns | Major concerns | No concerns | No concerns | Low | "Imprecision" |
| Antidepressants:Vitamin B12 | 1 | Some concerns | Low risk | No concerns | Major concerns | No concerns | No concerns | Very low | "Within-study bias","Imprecision" |
| Antiepileptics:Antiepileptics+Antidepressants | 1 | No concerns | Low risk | No concerns | Major concerns | No concerns | No concerns | Low | "Imprecision" |
| Antiepileptics:Placebo | 3 | Some concerns | Low risk | No concerns | Major concerns | No concerns | No concerns | Very low | "Within-study bias","Imprecision" |
| Crocin:Placebo | 1 | No concerns | Low risk | No concerns | Major concerns | No concerns | No concerns | Low | "Imprecision" |
| Nabiximols:Placebo | 1 | No concerns | Low risk | No concerns | Major concerns | No concerns | No concerns | Low | "Imprecision" |
| Opioids:Opioids+Antiepileptics | 3 | Some concerns | Low risk | No concerns | Major concerns | No concerns | No concerns | Very low | "Within-study bias","Imprecision" |
| Opioids:Opioids+Neurotropin | 2 | Some concerns | Low risk | No concerns | Some concerns | Some concerns | No concerns | Very low | "Within-study bias","Imprecision","Heterogeneity" |
| Opioids:Placebo | 1 | No concerns | Low risk | No concerns | No concerns | Some concerns | No concerns | Moderate | "Heterogeneity" |
| Opioids+Antidepressants:Opioids+Antiepileptics | 1 | No concerns | Low risk | No concerns | Major concerns | No concerns | No concerns | Low | "Imprecision" |
| Opioids+Antiepileptics:Opioids+Antiepileptics+Antidepressants | 1 | No concerns | Low risk | No concerns | No concerns | Some concerns | No concerns | Moderate | "Heterogeneity" |
| Opioids+Antiepileptics:Opioids+Antiepileptics+NSAIDs | 1 | Some concerns | Low risk | No concerns | Major concerns | No concerns | No concerns | Very low | "Within-study bias","Imprecision" |
| Antidepressants:Crocin | 0 | No concerns | Low risk | No concerns | Major concerns | No concerns | No concerns | Low | "Imprecision" |
| Antidepressants:Nabiximols | 0 | No concerns | Low risk | No concerns | Major concerns | No concerns | No concerns | Low | "Imprecision" |
| Antidepressants:Opioids | 0 | No concerns | Low risk | No concerns | Major concerns | No concerns | No concerns | Low | "Imprecision" |
| Antidepressants:Opioids+Antidepressants | 0 | No concerns | Low risk | No concerns | Major concerns | No concerns | No concerns | Low | "Imprecision" |
| Antidepressants:Opioids+Antiepileptics | 0 | No concerns | Low risk | No concerns | Major concerns | No concerns | No concerns | Low | "Imprecision" |
| Antidepressants:Opioids+Antiepileptics+Antidepressants | 0 | No concerns | Low risk | No concerns | Some concerns | Some concerns | No concerns | Low | "Imprecision","Heterogeneity" |
| Antidepressants:Opioids+Antiepileptics+NSAIDs | 0 | No concerns | Low risk | No concerns | Major concerns | No concerns | No concerns | Low | "Imprecision" |
| Antidepressants:Opioids+Neurotropin | 0 | No concerns | Low risk | No concerns | Major concerns | No concerns | No concerns | Low | "Imprecision" |
| Antiepileptics:Crocin | 0 | No concerns | Low risk | No concerns | Major concerns | No concerns | No concerns | Low | "Imprecision" |
| Antiepileptics:Nabiximols | 0 | No concerns | Low risk | No concerns | No concerns | Major concerns | No concerns | Low | "Heterogeneity" |
| Antiepileptics:Opioids | 0 | No concerns | Low risk | No concerns | No concerns | No concerns | No concerns | High |  |
| Antiepileptics:Opioids+Antidepressants | 0 | No concerns | Low risk | No concerns | Major concerns | No concerns | No concerns | Low | "Imprecision" |
| Antiepileptics:Opioids+Antiepileptics | 0 | Some concerns | Low risk | No concerns | Major concerns | No concerns | No concerns | Very low | "Within-study bias","Imprecision" |
| Antiepileptics:Opioids+Antiepileptics+Antidepressants | 0 | No concerns | Low risk | No concerns | No concerns | No concerns | No concerns | High |  |
| Antiepileptics:Opioids+Antiepileptics+NSAIDs | 0 | Some concerns | Low risk | No concerns | Major concerns | No concerns | No concerns | Very low | "Within-study bias","Imprecision" |
| Antiepileptics:Opioids+Neurotropin | 0 | Some concerns | Low risk | No concerns | Major concerns | No concerns | No concerns | Very low | "Within-study bias","Imprecision" |
| Antiepileptics:Vitamin B12 | 0 | Some concerns | Low risk | No concerns | Major concerns | No concerns | No concerns | Very low | "Within-study bias","Imprecision" |
| Antiepileptics+Antidepressants:Crocin | 0 | No concerns | Low risk | No concerns | Major concerns | No concerns | No concerns | Low | "Imprecision" |
| Antiepileptics+Antidepressants:Nabiximols | 0 | No concerns | Low risk | No concerns | Major concerns | No concerns | No concerns | Low | "Imprecision" |
| Antiepileptics+Antidepressants:Opioids | 0 | No concerns | Low risk | No concerns | Major concerns | No concerns | No concerns | Low | "Imprecision" |
| Antiepileptics+Antidepressants:Opioids+Antidepressants | 0 | No concerns | Low risk | No concerns | Major concerns | No concerns | No concerns | Low | "Imprecision" |
| Antiepileptics+Antidepressants:Opioids+Antiepileptics | 0 | No concerns | Low risk | No concerns | Major concerns | No concerns | No concerns | Low | "Imprecision" |
| Antiepileptics+Antidepressants:Opioids+Antiepileptics+Antidepressants | 0 | No concerns | Low risk | No concerns | Major concerns | No concerns | No concerns | Low | "Imprecision" |
| Antiepileptics+Antidepressants:Opioids+Antiepileptics+NSAIDs | 0 | No concerns | Low risk | No concerns | Major concerns | No concerns | No concerns | Low | "Imprecision" |
| Antiepileptics+Antidepressants:Opioids+Neurotropin | 0 | No concerns | Low risk | No concerns | Major concerns | No concerns | No concerns | Low | "Imprecision" |
| Antiepileptics+Antidepressants:Placebo | 0 | No concerns | Low risk | No concerns | Major concerns | No concerns | No concerns | Low | "Imprecision" |
| Antiepileptics+Antidepressants:Vitamin B12 | 0 | No concerns | Low risk | No concerns | Major concerns | No concerns | No concerns | Low | "Imprecision" |
| Crocin:Nabiximols | 0 | No concerns | Low risk | No concerns | Major concerns | No concerns | No concerns | Low | "Imprecision" |
| Crocin:Opioids | 0 | No concerns | Low risk | No concerns | Major concerns | No concerns | No concerns | Low | "Imprecision" |
| Crocin:Opioids+Antidepressants | 0 | No concerns | Low risk | No concerns | Major concerns | No concerns | No concerns | Low | "Imprecision" |
| Crocin:Opioids+Antiepileptics | 0 | No concerns | Low risk | No concerns | Major concerns | No concerns | No concerns | Low | "Imprecision" |
| Crocin:Opioids+Antiepileptics+Antidepressants | 0 | No concerns | Low risk | No concerns | Major concerns | No concerns | No concerns | Low | "Imprecision" |
| Crocin:Opioids+Antiepileptics+NSAIDs | 0 | No concerns | Low risk | No concerns | Major concerns | No concerns | No concerns | Low | "Imprecision" |
| Crocin:Opioids+Neurotropin | 0 | No concerns | Low risk | No concerns | Major concerns | No concerns | No concerns | Low | "Imprecision" |
| Crocin:Vitamin B12 | 0 | No concerns | Low risk | No concerns | Major concerns | No concerns | No concerns | Low | "Imprecision" |
| Nabiximols:Opioids | 0 | No concerns | Low risk | No concerns | Major concerns | No concerns | No concerns | Low | "Imprecision" |
| Nabiximols:Opioids+Antidepressants | 0 | No concerns | Low risk | No concerns | Major concerns | No concerns | No concerns | Low | "Imprecision" |
| Nabiximols:Opioids+Antiepileptics | 0 | No concerns | Low risk | No concerns | Major concerns | No concerns | No concerns | Low | "Imprecision" |
| Nabiximols:Opioids+Antiepileptics+Antidepressants | 0 | No concerns | Low risk | No concerns | Major concerns | No concerns | No concerns | Low | "Imprecision" |
| Nabiximols:Opioids+Antiepileptics+NSAIDs | 0 | No concerns | Low risk | No concerns | Major concerns | No concerns | No concerns | Low | "Imprecision" |
| Nabiximols:Opioids+Neurotropin | 0 | No concerns | Low risk | No concerns | Major concerns | No concerns | No concerns | Low | "Imprecision" |
| Nabiximols:Vitamin B12 | 0 | No concerns | Low risk | No concerns | Major concerns | No concerns | No concerns | Low | "Imprecision" |
| Opioids:Opioids+Antidepressants | 0 | No concerns | Low risk | No concerns | Major concerns | No concerns | No concerns | Low | "Imprecision" |
| Opioids:Opioids+Antiepileptics+Antidepressants | 0 | No concerns | Low risk | No concerns | Major concerns | No concerns | No concerns | Low | "Imprecision" |
| Opioids:Opioids+Antiepileptics+NSAIDs | 0 | Some concerns | Low risk | No concerns | Major concerns | No concerns | No concerns | Very low | "Within-study bias","Imprecision" |
| Opioids:Vitamin B12 | 0 | No concerns | Low risk | No concerns | Major concerns | No concerns | No concerns | Low | "Imprecision" |
| Opioids+Antidepressants:Opioids+Antiepileptics+Antidepressants | 0 | No concerns | Low risk | No concerns | Major concerns | No concerns | No concerns | Low | "Imprecision" |
| Opioids+Antidepressants:Opioids+Antiepileptics+NSAIDs | 0 | Some concerns | Low risk | No concerns | Major concerns | No concerns | No concerns | Very low | "Within-study bias","Imprecision" |
| Opioids+Antidepressants:Opioids+Neurotropin | 0 | Some concerns | Low risk | No concerns | Major concerns | No concerns | No concerns | Very low | "Within-study bias","Imprecision" |
| Opioids+Antidepressants:Placebo | 0 | No concerns | Low risk | No concerns | Major concerns | No concerns | No concerns | Low | "Imprecision" |
| Opioids+Antidepressants:Vitamin B12 | 0 | No concerns | Low risk | No concerns | Major concerns | No concerns | No concerns | Low | "Imprecision" |
| Opioids+Antiepileptics:Opioids+Neurotropin | 0 | Some concerns | Low risk | No concerns | Major concerns | No concerns | No concerns | Very low | "Within-study bias","Imprecision" |
| Opioids+Antiepileptics:Placebo | 0 | No concerns | Low risk | No concerns | Major concerns | No concerns | No concerns | Low | "Imprecision" |
| Opioids+Antiepileptics:Vitamin B12 | 0 | No concerns | Low risk | No concerns | Major concerns | No concerns | No concerns | Low | "Imprecision" |
| Opioids+Antiepileptics+Antidepressants:Opioids+Antiepileptics+NSAIDs | 0 | Some concerns | Low risk | No concerns | Some concerns | Some concerns | No concerns | Very low | "Within-study bias","Imprecision","Heterogeneity" |
| Opioids+Antiepileptics+Antidepressants:Opioids+Neurotropin | 0 | Some concerns | Low risk | No concerns | Major concerns | No concerns | No concerns | Very low | "Within-study bias","Imprecision" |
| Opioids+Antiepileptics+Antidepressants:Placebo | 0 | No concerns | Low risk | No concerns | No concerns | Some concerns | No concerns | Moderate | "Heterogeneity" |
| Opioids+Antiepileptics+Antidepressants:Vitamin B12 | 0 | No concerns | Low risk | No concerns | No concerns | Major concerns | No concerns | Low | "Heterogeneity" |
| Opioids+Antiepileptics+NSAIDs:Opioids+Neurotropin | 0 | Some concerns | Low risk | No concerns | Major concerns | No concerns | No concerns | Very low | "Within-study bias","Imprecision" |
| Opioids+Antiepileptics+NSAIDs:Placebo | 0 | Some concerns | Low risk | No concerns | Major concerns | No concerns | No concerns | Very low | "Within-study bias","Imprecision" |
| Opioids+Antiepileptics+NSAIDs:Vitamin B12 | 0 | Some concerns | Low risk | No concerns | Major concerns | No concerns | No concerns | Very low | "Within-study bias","Imprecision" |
| Opioids+Neurotropin:Placebo | 0 | Some concerns | Low risk | No concerns | Major concerns | No concerns | No concerns | Very low | "Within-study bias","Imprecision" |
| Opioids+Neurotropin:Vitamin B12 | 0 | Some concerns | Low risk | No concerns | Major concerns | No concerns | No concerns | Very low | "Within-study bias","Imprecision" |
| Placebo:Vitamin B12 | 0 | Some concerns | Low risk | No concerns | Major concerns | No concerns | No concerns | Very low | "Within-study bias","Imprecision" |

**Table S6D CINeMA for overall adverse events**

| Comparison | Number of studies | Within-study bias | Reporting bias | Indirectness | Imprecision | Heterogeneity | Incoherence | Confidence rating | Reason(s) for downgrading |
| --- | --- | --- | --- | --- | --- | --- | --- | --- | --- |
| Anesthetics:Placebo | 3 | No concerns | Low risk | No concerns | Major concerns | No concerns | No concerns | Low | "Imprecision" |
| Antidepressants:Placebo | 1 | No concerns | Low risk | No concerns | Major concerns | No concerns | No concerns | Low | "Imprecision" |
| Antiepileptics:Opioids | 1 | No concerns | Low risk | No concerns | No concerns | Some concerns | No concerns | Moderate | "Heterogeneity" |
| Antiepileptics:Placebo | 4 | Some concerns | Low risk | No concerns | Some concerns | Some concerns | No concerns | Very low | "Within-study bias","Imprecision","Heterogeneity" |
| Costus:Placebo | 1 | No concerns | Low risk | No concerns | Major concerns | No concerns | No concerns | Low | "Imprecision" |
| Anesthetics+antidepressants:Placebo | 1 | No concerns | Low risk | No concerns | Major concerns | No concerns | No concerns | Low | "Imprecision" |
| Opioids+antidepressants:Opioids+antiepileptics | 1 | No concerns | Low risk | No concerns | Major concerns | No concerns | No concerns | Low | "Imprecision" |
| Opioids+antiepileptics:Opioids | 1 | Some concerns | Low risk | No concerns | Some concerns | Some concerns | No concerns | Very low | "Within-study bias","Imprecision","Heterogeneity" |
| Opioids+neurotropin:Opioids | 1 | Some concerns | Low risk | No concerns | Major concerns | No concerns | No concerns | Very low | "Within-study bias","Imprecision" |
| Opioids:Placebo | 1 | No concerns | Low risk | No concerns | No concerns | Some concerns | No concerns | Moderate | "Heterogeneity" |
| Placebo:Spicamycin Derivative | 1 | No concerns | Low risk | No concerns | Major concerns | No concerns | No concerns | Low | "Imprecision" |
| Anesthetics:Antidepressants | 0 | No concerns | Low risk | No concerns | Major concerns | No concerns | No concerns | Low | "Imprecision" |
| Anesthetics:Antiepileptics | 0 | No concerns | Low risk | No concerns | Major concerns | No concerns | No concerns | Low | "Imprecision" |
| Anesthetics:Costus | 0 | No concerns | Low risk | No concerns | Major concerns | No concerns | No concerns | Low | "Imprecision" |
| Anesthetics:Anesthetics+antidepressants | 0 | No concerns | Low risk | No concerns | Major concerns | No concerns | No concerns | Low | "Imprecision" |
| Anesthetics:Opioids+antidepressants | 0 | No concerns | Low risk | No concerns | Major concerns | No concerns | No concerns | Low | "Imprecision" |
| Anesthetics:Opioids+antiepileptics | 0 | No concerns | Low risk | No concerns | Major concerns | No concerns | No concerns | Low | "Imprecision" |
| Anesthetics:Opioids+neurotropin | 0 | No concerns | Low risk | No concerns | Major concerns | No concerns | No concerns | Low | "Imprecision" |
| Anesthetics:Opioids | 0 | No concerns | Low risk | No concerns | Major concerns | No concerns | No concerns | Low | "Imprecision" |
| Anesthetics:Spicamycin Derivative | 0 | No concerns | Low risk | No concerns | Major concerns | No concerns | No concerns | Low | "Imprecision" |
| Antidepressants:Antiepileptics | 0 | No concerns | Low risk | No concerns | Major concerns | No concerns | No concerns | Low | "Imprecision" |
| Antidepressants:Costus | 0 | No concerns | Low risk | No concerns | Major concerns | No concerns | No concerns | Low | "Imprecision" |
| Antidepressants:Anesthetics+antidepressants | 0 | No concerns | Low risk | No concerns | Major concerns | No concerns | No concerns | Low | "Imprecision" |
| Antidepressants:Opioids+antidepressants | 0 | No concerns | Low risk | No concerns | Major concerns | No concerns | No concerns | Low | "Imprecision" |
| Antidepressants:Opioids+antiepileptics | 0 | No concerns | Low risk | No concerns | Major concerns | No concerns | No concerns | Low | "Imprecision" |
| Antidepressants:Opioids+neurotropin | 0 | No concerns | Low risk | No concerns | Major concerns | No concerns | No concerns | Low | "Imprecision" |
| Antidepressants:Opioids | 0 | No concerns | Low risk | No concerns | No concerns | Some concerns | No concerns | Moderate | "Heterogeneity" |
| Antidepressants:Spicamycin Derivative | 0 | No concerns | Low risk | No concerns | Major concerns | No concerns | No concerns | Low | "Imprecision" |
| Antiepileptics:Costus | 0 | No concerns | Low risk | No concerns | Major concerns | No concerns | No concerns | Low | "Imprecision" |
| Antiepileptics:Anesthetics+antidepressants | 0 | No concerns | Low risk | No concerns | Major concerns | No concerns | No concerns | Low | "Imprecision" |
| Antiepileptics:Opioids+antidepressants | 0 | No concerns | Low risk | No concerns | Major concerns | No concerns | No concerns | Low | "Imprecision" |
| Antiepileptics:Opioids+antiepileptics | 0 | Some concerns | Low risk | No concerns | Major concerns | No concerns | No concerns | Very low | "Imprecision" |
| Antiepileptics:Opioids+neurotropin | 0 | Some concerns | Low risk | No concerns | Major concerns | No concerns | No concerns | Very low | "Imprecision" |
| Antiepileptics:Spicamycin Derivative | 0 | No concerns | Low risk | No concerns | Major concerns | No concerns | No concerns | Low | "Imprecision" |
| Costus:Anesthetics+antidepressants | 0 | No concerns | Low risk | No concerns | Major concerns | No concerns | No concerns | Low | "Imprecision" |
| Costus:Opioids+antidepressants | 0 | No concerns | Low risk | No concerns | Major concerns | No concerns | No concerns | Low | "Imprecision" |
| Costus:Opioids+antiepileptics | 0 | No concerns | Low risk | No concerns | Major concerns | No concerns | No concerns | Low | "Imprecision" |
| Costus:Opioids+neurotropin | 0 | No concerns | Low risk | No concerns | Major concerns | No concerns | No concerns | Low | "Imprecision" |
| Costus:Opioids | 0 | No concerns | Low risk | No concerns | Major concerns | No concerns | No concerns | Low | "Imprecision" |
| Costus:Spicamycin Derivative | 0 | No concerns | Low risk | No concerns | Major concerns | No concerns | No concerns | Low | "Imprecision" |
| Anesthetics+antidepressants:Opioids+antidepressants | 0 | No concerns | Low risk | No concerns | Major concerns | No concerns | No concerns | Low | "Imprecision" |
| Anesthetics+antidepressants:Opioids+antiepileptics | 0 | No concerns | Low risk | No concerns | Major concerns | No concerns | No concerns | Low | "Imprecision" |
| Anesthetics+antidepressants:Opioids+neurotropin | 0 | No concerns | Low risk | No concerns | Major concerns | No concerns | No concerns | Low | "Imprecision" |
| Anesthetics+antidepressants:Opioids | 0 | No concerns | Low risk | No concerns | No concerns | Major concerns | No concerns | Low | "Heterogeneity" |
| Anesthetics+antidepressants:Spicamycin Derivative | 0 | No concerns | Low risk | No concerns | Major concerns | No concerns | No concerns | Low | "Imprecision" |
| Opioids+antidepressants:Opioids+neurotropin | 0 | Some concerns | Low risk | No concerns | Major concerns | No concerns | No concerns | Very low | "Within-study bias","Imprecision" |
| Opioids+antidepressants:Opioids | 0 | Some concerns | Low risk | No concerns | Major concerns | No concerns | No concerns | Very low | "Within-study bias","Imprecision" |
| Opioids+antidepressants:Placebo | 0 | No concerns | Low risk | No concerns | Major concerns | No concerns | No concerns | Low | "Imprecision" |
| Opioids+antidepressants:Spicamycin Derivative | 0 | No concerns | Low risk | No concerns | Major concerns | No concerns | No concerns | Low | "Imprecision" |
| Opioids+antiepileptics:Opioids+neurotropin | 0 | Some concerns | Low risk | No concerns | Major concerns | No concerns | No concerns | Very low | "Within-study bias","Imprecision" |
| Opioids+antiepileptics:Placebo | 0 | Some concerns | Low risk | No concerns | Major concerns | No concerns | No concerns | Very low | "Within-study bias","Imprecision" |
| Opioids+antiepileptics:Spicamycin Derivative | 0 | No concerns | Low risk | No concerns | Major concerns | No concerns | No concerns | Low | "Imprecision" |
| Opioids+neurotropin:Placebo | 0 | Some concerns | Low risk | No concerns | Major concerns | No concerns | No concerns | Very low | "Within-study bias","Imprecision" |
| Opioids+neurotropin:Spicamycin Derivative | 0 | No concerns | Low risk | No concerns | Major concerns | No concerns | No concerns | Low | "Imprecision" |
| Opioids:Spicamycin Derivative | 0 | No concerns | Low risk | No concerns | Some concerns | Some concerns | No concerns | Low | "Imprecision","Heterogeneity" |
